# Safety and clinical activity with an anti-PD-1 antibody JS001 in advanced melanoma or urologic cancer patients

**DOI:** 10.1186/s13045-018-0693-2

**Published:** 2019-01-14

**Authors:** Bixia Tang, Xieqiao Yan, Xinan Sheng, Lu Si, Chuanliang Cui, Yan Kong, Lili Mao, Bin Lian, Xue Bai, Xuan Wang, Siming Li, Li Zhou, Jiayi Yu, Jie Dai, Kai Wang, Jinwei Hu, Lihou Dong, Haifeng Song, Hai Wu, Hui Feng, Sheng Yao, Zhihong Chi, Jun Guo

**Affiliations:** 1 Key Laboratory of Carcinogenesis and Translational Research (Ministry of Education/Beijing), Department of Renal Cancer and Melanoma, Peking University Cancer Hospital & Institute, Collaborative Innovation Center for Cancer Medicine, Beijing, China; 2 OrigiMed, Shanghai, China; 3 Department of Pharmacology and Toxicology, Beijing Institute of Radiation Medicine, Beijing, China; 4 Shanghai Junshi Biosciences Co., Ltd., Shanghai, China

**Keywords:** PD-1, Monoclonal antibody, JS001, Melanoma, Urothelial cancer, Renal cell carcinoma

## Abstract

**Background:**

JS001, a humanized IgG_4_ monoclonal antibody against the programmed death-1 (PD-1) receptor, blocks the interaction of PD-1 with its ligands and promotes T cell activation in preclinical studies. This phase I study is designed to evaluate the safety, tolerability, and clinical activity of JS001 in advanced melanoma or urologic cancer patients who are refractory to standard systemic therapy.

**Patients and methods:**

In the dose escalation cohorts, subjects initially received a single-dose, intravenous infusion of JS001, and were followed for 28 days followed by multi-dose infusions every 2 weeks. In the dose expansion cohorts, subjects received multi-dose infusions every 2 weeks. Clinical response was evaluated after each 8-week treatment cycle according to RECIST v1.1 criteria.

**Results:**

Thirty-six subjects diagnosed with advanced melanoma (*n* = 22), urothelial cancer (UC) (*n* = 8), or renal cell cancer (RCC) (*n* = 6) were enrolled. Melanoma subjects included 14 acral and 4 mucosal subtypes. JS001 was well tolerated, and no dose-limiting toxicity was observed. By the safety data cutoff date, 100% of subjects had treatment-related adverse events (TRAE) with most adverse events being grade 1 or 2, and ≥ grade 3 TRAEs occurred in 36%. Among all 36 subjects, 1 confirmed complete response (acral melanoma), 7 confirmed partial responses (2 acral melanoma, 1 mucosal melanoma, 2 UC, and 2 RCC), and 10 stable disease were observed, for an objective response rate of 22.2% (95% CI, 10.1 to 39.2), and a disease control rate of 50.0% (95% CI, 32.9 to 67.1). Clinical responses were correlated with PD-L1 expression on tumor cells, the presence of tumor infiltrating lymphocytes (TIL), baseline tumor volume, ECOG performance status, serum LDH levels, high percentage of activated CD8+ T cells and CD3− CD16+ CD54+ NK cells in the peripheral blood as well as tumor mutational burden (TMB).

**Conclusion:**

JS001 was well tolerated and demonstrated promising anti-tumor activity in UC and RCC as well as in previously underexplored acral and mucosal melanoma subtypes. Subjects with an immune-active profile in the tumor microenvironment or in peripheral blood responded favorably to JS001 treatment. The completion of the current phase I study has led to the initiation of the first prospective anti-PD-1 registration trial in Asia focusing on acral and mucosal melanoma subtypes, representative of the regional disease epidemiology.

**Trial registration:**

Clinical Trial ID: NCT02836795, registered July 19, 2016, retrospectively registered.

**Electronic supplementary material:**

The online version of this article (10.1186/s13045-018-0693-2) contains supplementary material, which is available to authorized users.

## Background

Immune evasion is one of the hallmarks of cancer. Tumor-specific T lymphocytes are often present in the tumor microenvironment, in the draining lymph nodes, and in the periphery, but are generally unable to control tumor progression due to a network of immunosuppressive mechanisms present in the tumor microenvironment [1–3]. CD8^+^ tumor infiltrating T lymphocytes (TILs) often express activation induced inhibitory receptors including CTLA-4 and PD-1 [4], while tumor cells frequently express immunosuppressive ligands, including PD-1 ligand 1 (PD-L1, also known as B7-H1 or CD274), that suppress T cell activation and effector function [5]. Among the suppressive mechanisms, PD-1 and its ligands have emerged as a crucial pathway exploited by tumor cells to inhibit activated T cells in the tumor microenvironment [3].

PD-1 is an inhibitory member of the CD28 receptor family expressed on activated B and T lymphocytes and myeloid cells [6, 7]. Interaction of PD-1 and B7 family ligands PD-L1 and PD-L2 delivers inhibitory signals that contribute to the establishment and maintenance of peripheral immune tolerance [8, 9]. PD-L1, the primary PD-1 ligand in the peripheral tissue, is overexpressed in many solid tumors, and PD-L1 expression has been identified as a negative prognostic biomarker in various cancers associated with poor survival [5, 10].

In the past decade, PD-1/PD-L1 pathway blockade has been proven to be an effective approach in inducing durable anti-tumor responses in various cancer indications. Monoclonal antibodies (mAbs) that block the PD-1/PD-L1 pathway can enhance tumor specific T cell activation and effector function, reduce tumor burden, and improve survival [11, 12]. From year 2014 to 2017, FDA has approved two anti-PD-1 mAbs (nivolumab and pembrolizumab) and three anti-PD-L1 mAbs (atezolizumab, avelumab, and durvalumab) for human malignancies. Melanoma was the first indication approved for both nivolumab and pembrolizumab in 2014 [13, 14]. Melanoma has long been regarded as an immunogenic cancer type due to frequently observed lymphocyte infiltration into the tumor, as well as clinical response to immunotherapy of high dose IL-2 [15]. Mechanistically, chronic sun exposure-associated ultraviolet (UV)-induced DNA damage is the leading cause for melanoma in the western population. Chronic sun damage (CSD) melanoma accounts for 95% of cutaneous melanoma in the USA and other western countries [16]. In contrast, acral lentiginous melanoma (ALM) (~ 50%) and mucosal melanoma (MM) (~ 20%) are the two most common melanoma subgroups in Asia [17]. Neither ALM nor MM are associated with chronic UV exposure and thus harbor much fewer DNA mutations [18]. Retrospective studies showed immunotherapies were less effective treating ALM and MM when compared with CSD melanoma, presumably due to their low immunogenic properties and different molecular mechanisms for tumorigenesis [19].

JS001 is a humanized IgG_4_ mAb specific for human PD-1 [20]. Here, we report the first-in-human dose-escalation study of JS001 conducted at Peking University Cancer Hospital in metastatic solid tumor subjects refractory to standard systemic treatment, including 22 melanoma, 8 urothelial carcinoma (UC), and 6 renal cell carcinoma (RCC) subjects. Among 22 melanoma subjects, 14 (64%) were acral and 4 (18%) were mucosal melanoma subtypes. JS001 had an acceptable overall safety profile up to 10 mg/kg Q2W with no novel safety signal identified when compared with marketed drugs in the same class. Objective clinical responses were observed at every dose level and in all three indications.

## Patients and methods

### JS001

JS001, also known as TAB001 or toripalimab, is a humanized IgG_4_κ mAb specific for human PD-1 receptor and blocks interactions of PD-1 with its ligands, PD-L1 and PD-L2. JS001 contains the complementary-determining regions (CDR) of a murine antibody that binds to human PD-1 and human framework regions with limited back-mutations to the parental murine sequences. A serine to proline substitution was introduced at amino acid 228 (S228P) to minimize Fab arm exchange of the IgG_4_ molecule. JS001 is produced by recombinant DNA technology in a Chinese Hamster Ovary (CHO) mammalian cell expression system (LONZA).

### Patients

Eligible subjects were between 18 to 70 years old with metastatic melanoma or urologic cancers who were refractory to standard systemic treatment. Subjects had measurable disease per RECIST v1.1, Eastern Cooperative Oncology Group (ECOG) performance status of 0 or 1, adequate organ and marrow function, willingness to provide consent for biopsy samples, and no history of autoimmune diseases or ongoing infections. Concurrent antineoplastic therapies, systemic steroid treatment, and prior immunotherapies with anti-CTLA4, anti-PD-1, and anti-PD-L1 were not allowed. Prior monoclonal antibody treatments were allowed but not within 3 months of the start of JS001 treatment. Other prior non-antibody immunotherapies were also allowed but not within 4 weeks of JS001 treatment.

### Study design

This is a single-center, phase 1, open-label, 2-part (part A dose-escalation and part B dose-expansion) study to evaluate the safety, tolerability, pharmacokinetics (PK), pharmacodynamics, immunogenicity, and antitumor activity of JS001, administered via intravenous (IV) infusion on an every 2-week (Q2W) dosing schedule in adult subjects with advanced melanoma or urologic cancers refractory to standard therapy (Clinical Trial ID: NCT02836795). This phase I study was approved by Peking University Cancer Hospital institutional review board.

In part A, the study could enroll up to 18 subjects with advanced melanoma or urologic cancers that were immunotherapy-naïve (no prior exposure to immunotherapy such as but not limited to anti-CTLA-4, anti-PD-1, or anti-PD-L1 antibodies). The planned cohorts in part A were 1 mg/kg, 3 mg/kg, and 10 mg/kg Q2W. A minimum of 3 subjects were initially enrolled at each dose level. Sequential subjects in a cohort received a single 60-min IV infusion of JS001 and were evaluated for toxicity for 28 days. DLT was defined as a treatment-related grade 3 or above AE or laboratory abnormality occurring within 28 days after the first dose. Provided there was no DLT in the initial 3 subjects, escalation to the next cohort could occur. If a DLT did occur, that cohort would be expanded to a total of 6 subjects. After 28 days following the first dose, subjects continued to receive JS001 at the intended dose level Q2W, followed by radiologic evaluation every 8 weeks (Q8W). Responses were evaluated by investigators using the Response Evaluation Criteria in Solid Tumors (RECIST) v1.1 and Immune-related Response Evaluation Criteria In Solid Tumors (irRECIST). Subjects with progressive disease or an intolerant toxicity were taken off the study. Patients who initially developed progressive disease per RECISTv1.1 were allowed to continue therapy if investigator considered patients were benefiting from the treatment per irRECIST.

Any dose-escalation cohort that did not exceed the maximum-tolerated dose (MTD) could be expanded up to a maximum of 12 subjects in part B for further evaluation of safety, PK, pharmacodynamics, and clinical activity. In part B, subjects received JS001 through IV infusion at either 1, 3, or 10 mg/kg per dose Q2W, followed by radiologic evaluation Q8W until disease progression, development of intolerable toxicity, or voluntary withdrawal from the study. AEs are reported as of July 31, 2018. The latest survival follow-up was provided by July 3, 2018.

### Tumor biopsies and immunohistochemistry

Archival or fresh tumor biopsies were obtained from subjects prior to JS001 treatment. Detection of PD-L1 and CD8+ T cells by immunohistochemistry (IHC) staining of formalin-fixed paraffin-embedded (FFPE) tumor tissue samples was performed at Peking University Cancer Hospital. Spring Bioscience (Roche) rabbit anti-human PD-L1 mAb (Clone SP142, Cat No.: M4422) and CD8 antibody (Clone 4B11, BioRad, Cat# MCA 1817 T) were used for the PD-L1 CD8 double staining assay. Citrate buffer pH 6.0 was used for antigen retrieval, and a TSA plus biotin kit (Perkin Elmer) was used for signal amplification, followed by development with Peroxidase Substrate Kit for PD-L1 (red color), and ImmPRESS AP reagent and Blue substrate kit (Vector lab) for CD8 (blue color) staining. Stained tumor tissue was evaluated for PD-L1 expression on tumor cells (TC) by a certified pathologist. PD-L1-positive status was defined as the presence of membrane staining of any intensity in ≥ 1% of tumor cells.

Human tonsil tissue was used as dual positive and negative tissue control including positive tissue elements (moderate to strong PD-L1 staining in lymphocytes and macrophages in germinal centers, with diffuse staining in reticulated crypt epithelial cells) and negative tissue elements (PD-L1-negative immune cells in the inter-follicular regions with negative superficial squamous epithelium).

### Pharmacokinetics

Serum samples were collected 0.5 h prior to dosing and serially at the end of infusion (EOI), 0.5, 2, 6, 12, 24, 48, 96, 168, 336, 504, and 648 h after the first dose of JS001. An electrochemiluminescence (ECL) method was developed and validated for the detection and quantitation of JS001 in human serum, with lower limit of quantification (LLOQ) at 2.56 ng/mL. The capture reagent was biotinylated His-tag human PD-1 extracellular domain, and the detecting reagent was ruthenylated His-tag human PD-1. For all assays, the samples were incubated with labeled capture and detection reagents to allow for the formation of molecular complexes. The complexed samples were loaded into the wells of a streptavidin-coated carbon-electron plates (MSD). After unbound material was removed by washing, MSD read buffer was added and the bound complexes were detected by reading ECL signals using an MSD instrument. Standard curve was established with JS001 serially diluted in human serum. PK parameters for evaluation included area under the concentration-time curve (AUC), maximum observed concentration (*C*_max_), clearance (C_L_), volume of distribution (*V*_d_), and elimination half-life (t1/2).

### Immunogenicity

Serum sample was collected before the first dose of JS001 and every 28 days prior to dosing. Another ECL method was developed and validated for the detection and quantitation of anti-JS001 antibodies in human serum. In this assay, anti-drug antibodies (ADA) were complexed between biotinylated and ruthenylated JS001. The negative control was a human serum pool. Positive control samples were prepared by spiking the negative control serum pool with purified rabbit polyclonal anti-JS001 antibodies. For all assays, the samples were diluted at 1:10 in master mix and then incubated with labeled drug to allow for the formation of molecular complexes. The complexed samples were loaded into the wells of a streptavidin-coated carbon-electron plates (MSD). After unbound material was removed by washing, MSD read buffer was added and the bound complexes were detected by reading ECL signals using a MSD instrument. Samples that produced signals higher than the screening cut point (SCP) S/*N* = 1.05 were considered positive samples and were subject to the confirmation process in a competition assay in the presence of unlabeled JS001. The screening confirmation cut point (CCP) was determined to be 10.8%. If the response of the test sample was above CCP and confirmed, it was reported as an ADA-positive sample. The corresponding JS001 trough concentration was also evaluated in ADA-positive samples for neutralizing activity.

### Pharmacodynamics study of JS001 by PD-1 receptor occupancy

The pharmacodynamics study of JS001 was assessed by its ability to occupy its natural antigen, PD-1 receptor on peripheral T lymphocytes by flow cytometry, using subjects’ whole blood samples. PD-1 receptor occupancy (RO) by JS001 on target cells was evaluated during treatment. For subjects enrolled in part A (dose escalation cohorts), whole blood samples were collected by phlebotomy from each subject prior to the first dose (day 0), 24 h (day 1), 96 h (day 4), 168 h (day 7), and every week until day 28, and thereafter, every 2 weeks prior to the subsequent dose until day 126, and then every 28 days prior to the next dose until the end of the study. For subjects in part B (expansion cohorts), whole blood samples were collected prior to the first dose (day 0) and every 2 weeks prior to the subsequent dose until day 98, and thereafter, every 28 days prior to the next dose until the end of the study. All samples were analyzed within 48 h of collection. For each sample, 2 aliquots of 100 μL of blood sample were washed twice in 1 mL of staining buffer (1% FBS in PBS) before being incubated at room temperature (RT) for 15 min with either 5 μg of JS001 for total PD-1 or 5 μg of human IgG_4_ isotype control antibody (anti-KLH antibody) for on-board JS001 (PD-1 RO). After an extensive washing, both tubes were stained at RT for 30 min with a cocktail of mouse anti-human IgG_4_ pFc’-PE (Southern Biotech), FITC mouse anti-CD3ε (BD Biosciences), PerCP/Cy5.5 anti-human CD8a (BioLegend), and APC mouse anti-CD45RA (BD Biosciences). Red blood cells were lysed by incubating stained samples at RT for 15 min with FACS lysing solution (BD Biosciences). Samples were acquired and analyzed using a BD FACSCanto cytometer.

Flow cytometry data was analyzed using FlowJo software. T cells were gated based on positive CD3 staining and low side scatter (SSC) level. Doublets and cell clumps were excluded by gating on FSC-H versus FSC-A channels. CD8^+^ T cell subsets were further gated from the CD3^+^ singlet population. T cell activation status was analyzed by CD45RA expression level. Activated T cells were defined as CD3+ CD45RA negative cells. The PD-1 staining was analyzed in both CD3^+^CD8^+^CD45RA^−^ T cells and CD3^+^CD8^−^CD45RA^−^ T cells. Consistent gating was used for all samples. PD-1 RO was calculated as percentage of on-board JS001 staining over total PD-1 staining. Parameters evaluated included the mean fluorescence intensity (MFI) of JS001 staining and RO (%) in activated CD8^+^ T cells (CD3^+^CD8^+^CD45RA^−^) and CD8^−^ (CD4) T cells (CD3^+^CD8^−^CD45RA^−^). Occupancy above 80% was considered full RO. The results obtained were further analyzed using GraphPad PRISM 5.0 software.

### Genomic profiling and tumor mutational burden measurement

Comprehensive genomic profiling was performed by next-generation sequencing with a 450 cancer-related gene panel [21](Yuansu Cancer Panel, OrigiMed) on both FFPE tumor and paired peripheral blood samples obtained from 23 subjects. Briefly, genomic DNA was extracted from unstained FFPE slides with tumor content no less than 20% and was fragmented to ~ 250 bp by sonication. A DNA library was constructed using KAPA Hyper Prep Kit (KAPA Biosystems). Hybridization capture was conducted with a custom panel covering a total of 2.6 Mb of the human genome and paired-end sequencing (2 × 150 bp) was performed by following manufacturer’s protocols. Genomic alterations including single base substitution (SNV), short and long insertions/deletions (INDELs), copy number variants (CNV), and gene rearrangement and fusions were assessed. The tumor mutational burden (TMB) was estimated by analyzing somatic mutations including coding base substitution and INDELs per mega-base of the panel sequences examined. Driver gene mutations and known germline alterations in the single nucleotide polymorphism database (dbSNP) were excluded.

## Results

### Patients and treatments

From March 30, 2016, through December 26, 2016, 36 subjects with advanced metastatic melanoma (*n* = 22), renal cell carcinoma (*n* = 6), or urothelial carcinoma (*n* = 8) refractory to standard systemic therapy were enrolled in three dose escalating cohorts at 1 mg/kg (*n* = 3), 3 mg/kg (*n* = 4), and 10 mg/kg (*n* = 3) and three expansion cohorts at 1 mg/kg (*n* = 12), 3 mg/kg (*n* = 11), and 10 mg/kg (*n* = 3) (Table 1, Table 2, and Additional file 1: Figure S1). The median and average ages were 51 years old, with 21 (58.3%) male and 15 (41.7%) female subjects. Twenty (55.6%) subjects had an ECOG performance score of 1, and 16 (44.4%) subjects had an ECOG score of 0. The enrolled subjects were heavily pre-treated with a median of 3 prior lines of systemic treatment (range 1–11). One hundred percent of subjects had prior systemic chemotherapy. Among 22 melanoma subjects, 14 acral (63.6%), 4 mucosal (18.2%), 1 CSD (4.5%), and 3 non-CSD subtypes (13.6%) (1 chest lesion, 1 hip lesion, and 1 unknown primary lesion) were enrolled.

**Table 1:** Patient demographics

Characteristics	Specification	Value (%)
Age, years	Mean ± Std	51.0 ± 9.3
	Min~Max	28.1.70.1
Gender	Male	21 (58.3)
	Female	15 (41.7)
Tumor histology	Melanoma	22 (61.1)
	Urothelial carcinoma	8 (22.2)
	Renal cell carcinoma	6 (16.7)
ECOG PS	0	16 (44.4)
	1	20 (55.6)
PD-L1 expression*	Positive	16 (44.4)
	Negative	12 (33.3)
	NA	8 (22.2)
Prior therapies	Chemotherapy	36 (100.0)
	Surgery	34 (94.4)
	Radiation therapy	7 (19.4)

Age, gender, histology, ECOG performance score, PD-L1 expression on tumor biopsy by IHC staining, and prior therapies were summarized. *PD-L-positive status was defined as the presence of membrane staining of any intensity in ≥ 1% of tumor cells

**Table 2:** Tumor histology

Tumor histology	1 mg/kg	3 mg/kg	10 mg/kg	Total
Melanoma	10	10	2	22
Urothelial carcinoma	3	2	3	8
Renal cell carcinoma	2	3	1	6
Total	15	15	6	36

Total 36 subjects were enrolled in the trial, including 22 melanomas, 8 urothelial carcinomas, and 6 renal cell carcinomas in three JS001 dose cohorts of 1 mg/kg (*n* = 15), 3 mg/kg (*n* = 15), and 10 mg/kg (*n* = 6) given via intravenously infusion every 2 weeks (Q2W)

### Treatment-related toxicities

JS001 was well tolerated, and no DLT or infusion reactions were observed in the study. By the cutoff date of July 31, 2018, 19 months after the last subject was enrolled, subjects had received 2 to 49 doses of JS001 (12.4 doses on average). Average safety follow-up time was 5.8 months (range 0.5–22.9 months). One hundred percent of subjects had treatment-related adverse events (TRAE), but mostly were grade 1 or 2 (Table 3). Treatment-related serious adverse events (SAE) occurred in 5 (14%) subjects (Table 3 and Additional file 1: Table S1). Treatment was permanently terminated in 5 (14%) subjects due to AEs. Treatment was temporarily paused in 6 (16.7%) subjects due to AEs, and all 6 subjects re-initiated treatment after AEs were resolved. Two TRAEs leading to death were attributed to disease progression and were determined to be possibly unrelated to JS001 by the investigator.

**Table 3:** Summary of treatment-related adverse events (TRAE) in each cohort

	1 mg/kg (*n* = 15)		3 mg/kg (*n* = 15)		10 mg/kg (*n* = 6)		Total (*n* = 36)	
	Case	*N* (%)	Case	*N* (%)	Case	*N* (%)	Case	*N* (%)
Total TRAE	398	15 (100.00)	295	15 (100.00)	96	6 (100.00)	789	36 (100.00%)
TRAE ≥ grade 3	8	5 (33.33)	10	5 (33.33)	7	3 (50.00)	25	13 (36.11%)
Termination due to TRAE	0	0 (0.00)	0	0 (0.00)	0	0 (0.00)	0	0 (0.00%)
Pause due to TRAE	2	2 (13.33)	3	3 (20.00)	1	1 (16.67)	6	6 (16.67%)
Treatment-related SAE	3	2 (13.33)	1	1 (6.67)	3	2 (33.33)	7	5 (13.89%)
TRAE/SAE leading to death	0	0 (0.00)	0	0 (0.00)	2	2 (33.33)	2	2 (5.56%)

By the safety data cutoff date of July 31, 2018, 19 months after the last patient in, the total number and incidence rate of TRAE in each cohort, grade 3 and above TRAE, termination and pause due to TRAE, SAE and TRAE/SAE leading to death were listed. Two TRAEs (10 mg/kg cohort) that leading to death were attributed to disease progression and possibly unrelated to JS001 according to investigator’s evaluation. “Related,” “probably related,” “possibly related,” and “possibly not related” are all classified as “study drug/treatment related” in this trial

Grade 3 or above TRAEs occurred in 13 (36%) of subjects, including lipase increased (*n* = 4), anemia (*n* = 3), hypokalemia (*n* = 2), direct bilirubin (DBIL) increased (*n* = 2), and one occurrence each of ALT increased, amylase increased, proteinuria, creatine kinase increased, serum creatinine increased, hyperglycemia, low blood pressure, and kidney disease (Additional file 1: Table S2).

Common TRAEs included hyperglycemia (58%), proteinuria (50%), rash (44%), lipase increased (38%), anemia (33%), pruritus (25%), amylase increased (25%), leukopenia (25%), AST increased (25%), and ALT increased (22%) (Table 4). Immune-related AEs (irAEs) were of special interest as this class of drug promotes T cell activation and are known to cause autoimmune conditions. Observed irAEs included two hypothyroidisms (one grade 1 and one grade 2) and one hyperthyroidism (grade 1). The occurrence and severity of AEs/SAEs does not appear to be dose related. Overall, JS001 had an acceptable safety profile with no novel safety signal identified when compared with the two approved anti-PD-1 mAbs, nivolumab, and pembrolizumab.

**Table 4:** Common (≥ 20%) JS001 treatment-related adverse events from all subjects (*n* = 36)

Treatment-related AE	*N* (%)	Grade 1	2	3	4
Hyperglycemia	21 (58.33%)	19	1	1	0
Proteinuria	18 (50.00%)	14	3	1	0
Rash	16 (44.44%)	16	0	0	0
Thyroid stimulating hormone increased	16 (44.44%)	15	1	3	0
Lipase increased	14 (38.89%)	9	1	4	0
Fever	14 (38.89%)	13	1	0	0
Tri-iodothyronine free decreased	13 (36.11%)	12	1	0	0
Sinus tachycardia	12 (33.33%)	12	0	0	0
Anemia	12 (33.33%)	6	3	3	0
Leukocyturia	11 (30.56%)	11	0	0	0
Pruritus	9 (25.00%)	9	0	0	0
Leukopenia	9 (25.00%)	6	3	0	0
Amylase increased	9 (25.00%)	7	1	1	0
Thyroid hormone increases	9 (25.00%)	9	0	0	0
DBIL	9 (25.00%)	4	3	2	0
Hematuria	9 (25.00%)	9	0	0	0
AST increased	9 (25.00%)	9	0	0	0
Thyroid stimulating hormone decreased increased	9 (25.00%)	9	0	0	0
Hypochloremia	9 (25.00%)	9	0	0	0
White blood cell count increased	8 (22.22%)	8	0	0	0
ALT increased AAL	8 (22.22%)	7	0	1	0
TBIL increased	8 (22.22%)	6	2	0	0
Appetite decreased	8 (22.22%)	8	0	0	0
Fatigue	8 (22.22%)	7	1	0	0

By the safety data cutoff date of July 31, 2018, common TRAE total occurrence number (rate) and number in each grade were listed

### Antitumor activities

**Fig. 1:**
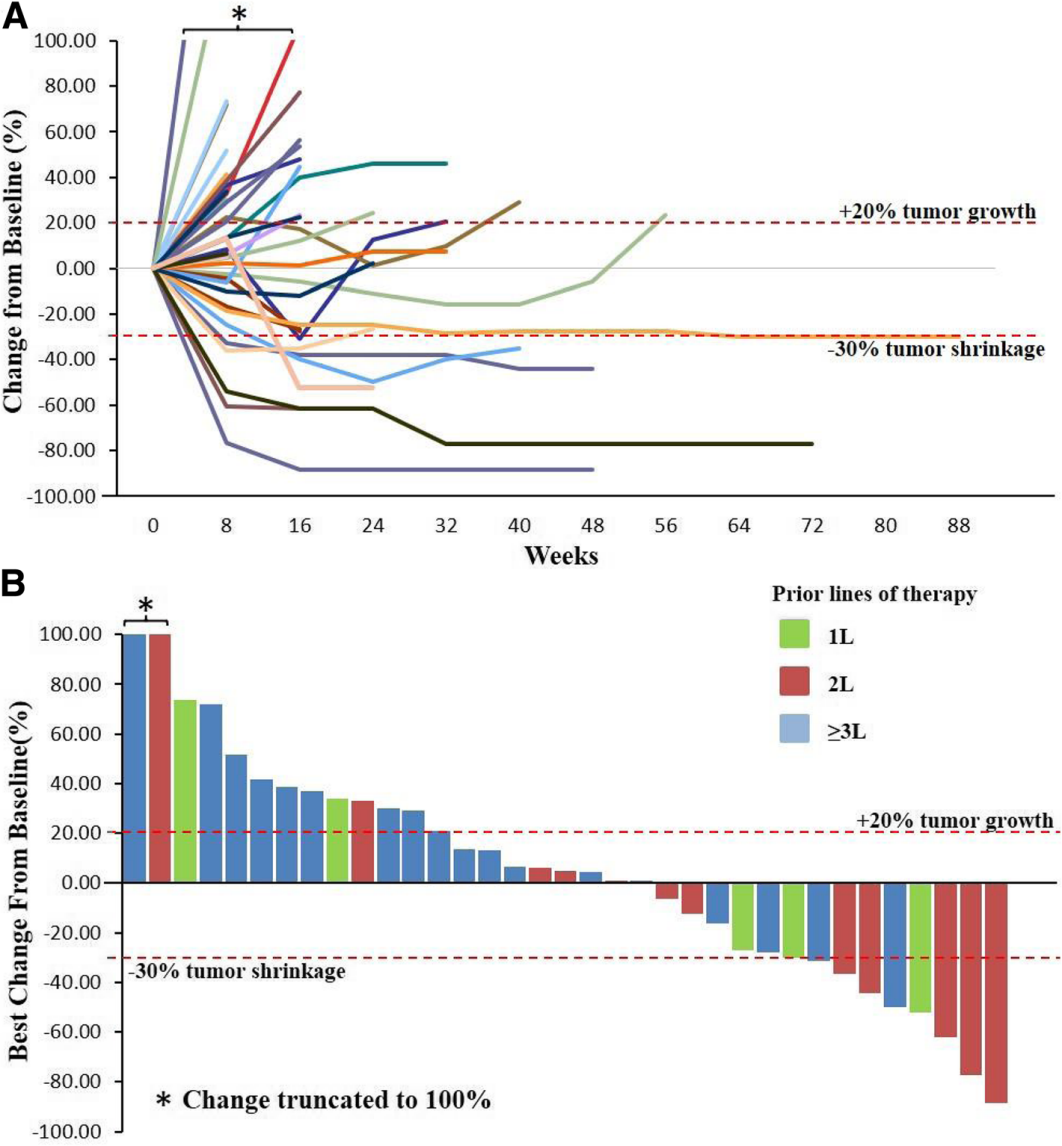
Antitumor activity of JS001. Clinical response was evaluated using Response Evaluation Criteria in Solid Tumors (RECIST) v1.1 by investigators every 8 weeks. **a** The percentage of sum of diameters of target lesions over baseline measurement during JS001 treatment is shown for each subject over time in the spider plot. Responses were durable in most patients, as the median duration of response was 5.6 months (range from 1.8 to 17.7+ months). **b** Waterfall plot of best percentage reduction in tumor burden from baseline is shown. Prior lines of treatment were marked by color for each subject. Seven out of 8 responding subjects had received at least two prior systemic treatments

Clinical response was evaluated using Response Evaluation Criteria in Solid Tumors (RECIST) v1.1 by investigators every 8 weeks. As of July 3, 2018, 1 acral melanoma subject from the 1 mg/kg expansion cohort dropped out of the trial voluntarily on day 15 after receiving two doses of JS001 and no post-treatment radiographic imaging evaluation was available from this subject. This subject was still included in the intention-to-treat (ITT) population for efficacy evaluation. Among all 36 enrolled subjects, 1 confirmed complete response (acral melanoma), 7 confirmed partial responses (2 acral melanoma, 1 mucosal melanoma, 2 UC, and 2 RCC), and 10 stable disease (including 1 unconfirmed partial response (PR) of UC) were observed, for an objective response rate (ORR) of 22.2% (95% CI, 10.1 to 39.2) and a DCR of 50.0% (95% CI, 32.9 to 67.1) (Table 5). Clinical responses were observed in every dose level and in all three cancer types. The best objective response from the 1, 3, and 10 mg/kg dose cohorts was 21.4% (including 1 unconfirmed PR), 26.7%, and 33.3%, while the DCR was 64.3%, 46.7%, and 33.3%, respectively. No apparent dose-related clinical efficacy was observed. Seven out of 8 responding subjects had received at least two prior systemic treatments. The change of tumor size (sum of diameters of target lesions) over time and the best response from baseline were shown in spider plot (Fig. 1a) and waterfall plot (Fig. 1b).

**Table 5:** JS001 clinical response was evaluated per RECIST v1.1 by investigators every 8 weeks

Histology	1 mg/kg (*n* = 15)	3 mg/kg (*n* = 15)	10 mg/kg (*n* = 6)	Total (*n* = 36)	ORR, DCR
Melanoma (*n* = 22)	1PR, 4SD	1CR, 2PR, 2SD	0PR	1CR, 3PR, 6SD	18.2%, 45.5%
UC (*n* = 8)	1PR, 2SD (1uPR)	1SD	1PR	2PR, 3SD	25.0%, 67.5%
RCC (*n* = 6)	1SD	1PR	1PR	2PR, 1SD	33.3%, 50.0%
Total	2PR, 7SD	1CR, 3PR, 3SD	2PR	1CR, 7PR, 10SD	22.2%, 50.0%
ORR, DCR	13.3%, 60.0%	26.7%, 46.7%	33.3%, 33.3%	22.2%, 50.0%	

As of July 31, 2018, among 36 enrolled subjects, 1 confirmed complete response (CR) (acral melanoma), 7 confirmed partial response (PR) (2 acral melanoma, 1 mucosal melanoma, 2 UC, and 2 RCC), and 10 confirmed stable disease (SD) (including 1 unconfirmed PR of UC) were observed, for an objective response rate (ORR) of 22.2% (95% CI, 10.1 to 39.2) and a disease control rate (DCR) of 50.0% (95% CI, 32.9 to 67.1)

The median time to response was 12 weeks (range 8 to 65.7 weeks), typical of immune checkpoint blockade therapy which requires time to elicit an anti-tumor effect. Notably, 1 UC subject maintained stable disease for 460 days until a PR was achieved. Responses were durable in most subjects as the median duration of response was 5.6 months (range from 1.8 to 17.7+ months).

Among 13 evaluable acral melanoma subjects, 1 confirmed complete response (CR), 2 confirmed PR, and 3 confirmed stable disease (SD) were achieved, for a 23.1% ORR and a 46.2% DCR. Meanwhile, 1 confirmed PR and 1 SD were observed in 4 mucosal melanoma subjects. The duration of response was 8.0, 8.4, 10.6, and 17.7+ months for 4 responding melanoma subjects who had received 2 to 5 lines of prior systemic treatment, indicating PD-1 blockade as a monotherapy could induce durable responses in heavily pretreated acral and mucosal melanoma subjects. One mucosal subject still had an ongoing response after receiving JS001 treatment for 19.5 months.

### Progression free survival (PFS) and overall survival (OS) analysis

**Fig. 2:**
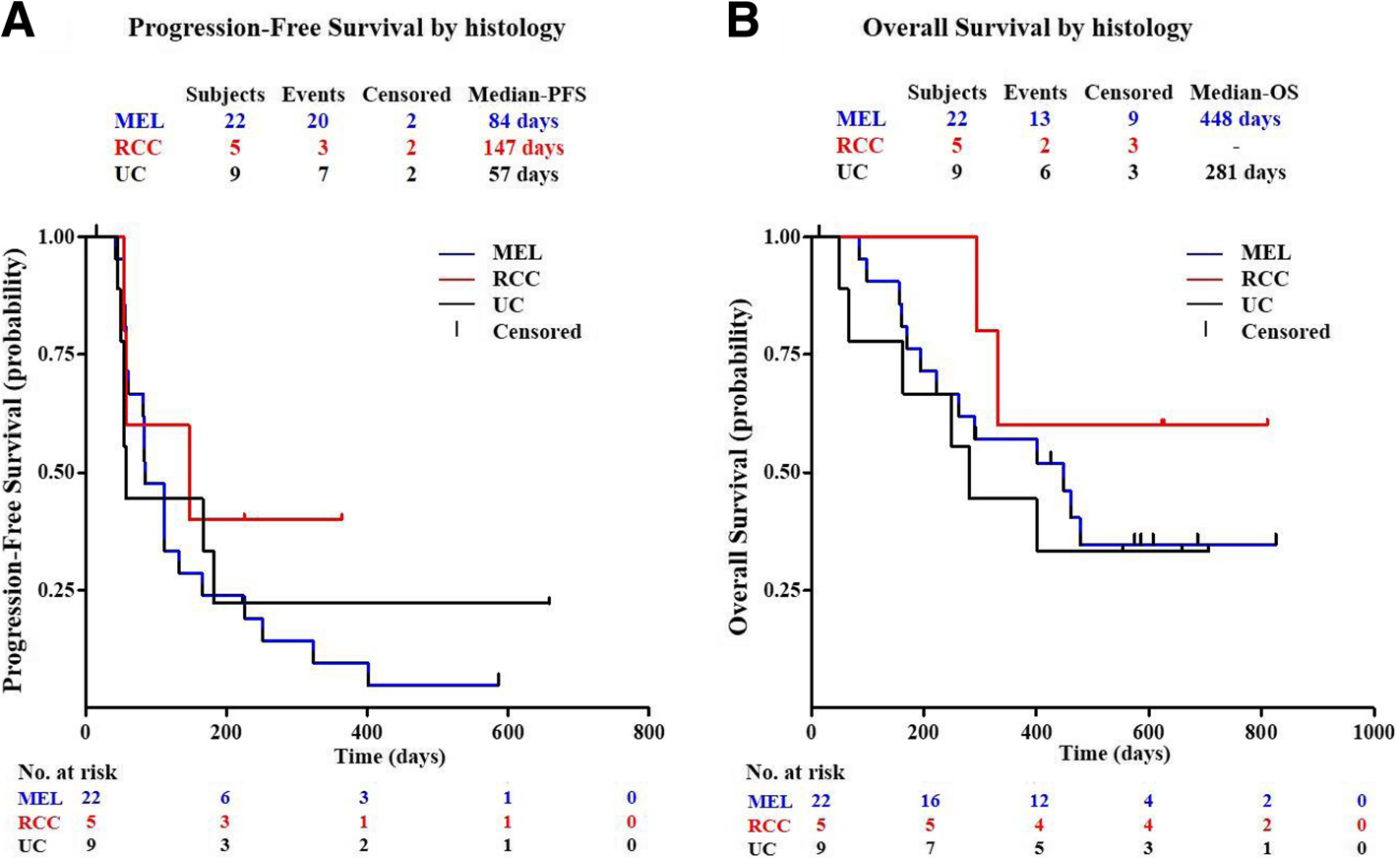
Survival after JS001 treatment. **a** Progression-free survival (PFS) of subjects by histology. **b** Overall survival (OS) of subjects by histology. Percentages of survival patients are shown at indicated time points. Numbers of patients at risk at indicated time points are shown below the *x*-axis

At the last survival follow-up on July 3, 2018, 21 subjects were deceased, 4 subjects were lost to follow-up, and 11 subjects were still alive. The median PFS was 2.8 months (range 0.5 to 21.9+ months). The median overall survival was 12.2 months (range 1.7 to 27.5+ months). There were no statistically significant differences by tumor histology in PFS and OS (Fig. 2), while RCC had the best median OS and PFS numerically. The 1-year survival rate was 50.0% for all subjects.

### Pharmacokinetics and immunogenicity

An ECL method was developed and validated for the detection and quantitation of JS001 concentration in human serum. JS001 serum concentration reached *C*_max_ about 1 h after infusion and was slowly eliminated from the circulation. JS001 exhibited a dose-dependent linear PK profile. Accumulation of JS001 was observed when given as a multi-dose infusion. The measured serum half-life of JS001 was 6.2 ± 1.3, 7.7 ± 3.5, and 9.8 ± 4.1 days respectively after a single infusion, and 9.5 ± 2.0, 16.5 ± 7.8, and 13.9 ± 5.5 days respectively after multi-dose infusions of 1 mg/kg, 3 mg/kg, and 10 mg/kg given IV Q2W (Additional file 1: Figure S2A).

The serum JS001 trough concentration plateaued after approximately five consecutive doses. The accumulation index was 1.57 ± 0.19, 2.25 ± 0.77, and 2.00 ± 0.54 for the 1, 3, and 10 mg/kg cohorts, respectively. The serum concentration of JS001 over time is shown in Additional file 1: Figure S2B. An in vitro study had previously shown that the IC_50_ of JS001 blocking PD-1 receptor ligand binding was 3 nmol/L (~ 0.5 μg/mL) [20]. The steady state trough concentration of JS001 in the trial was 8.9 ± 4.4, 37.8 ± 17.5, and 174.3 ± 95.6 μg/mL respectively for the 1 mg/kg, 3 mg/kg, and 10 mg/kg cohorts, indicating the PD-1 receptor will be fully occupied even at the lowest dose cohort of 1 mg/kg.

Serum samples were collected prior to the 1st dose of JS001 and every 28 days prior to dosing. The presence of JS001 ADA was determined using a validated Bridging-ECL method (Meso Scale Discovery, Inc.). No ADA-positive samples were detected in the 10 mg/kg cohort; 3 subjects in the 1 mg/kg and 4 subjects in 3 mg/kg cohort had ADA positivity detected. One subject in 3 mg/kg cohort had an ADA-positive sample before the first dose, reflecting a pre-existing condition. Accelerated JS001 serum clearance and reduced trough concentration coincided with the detection of ADA-positive signals in 1 subject in the 1 mg/kg cohort and 2 subjects in the 3 mg/kg cohort, indicating the presence of neutralizing ADA (8.3%, 3/36). These 3 subjects experienced progressive disease clinically, indicating neutralizing ADA might affect JS001 anti-tumor efficacy.

### Pharmacodynamic study of JS001 by PD-1 receptor occupancy and T cell activation status

**Fig. 3:**
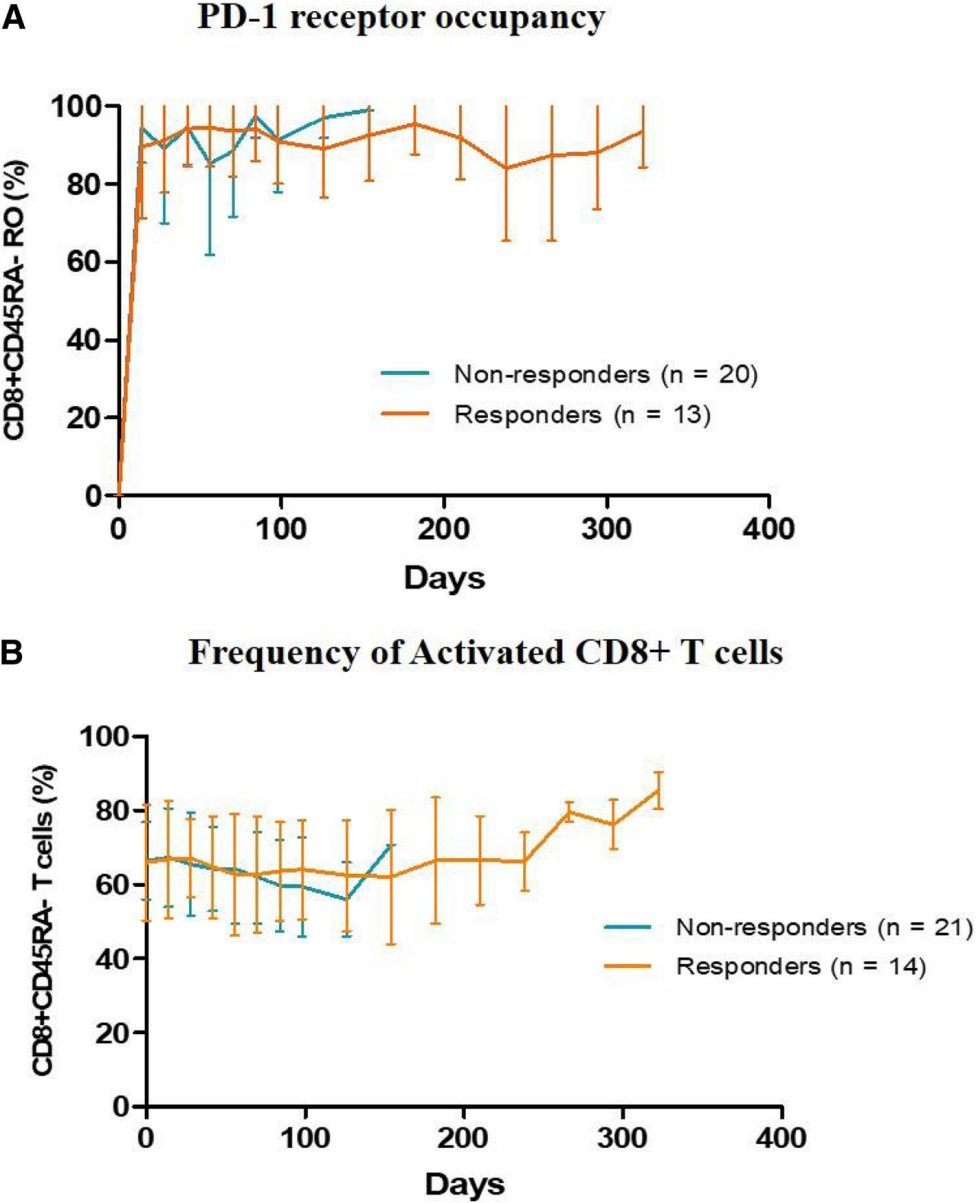
Pharmacodynamic readouts of JS001. **a** PD-1 receptor occupancy (RO) was determined as the percentage of receptor bound JS001 to total PD-1 on activated lymphocytes (CD3+ CD45RA−) from the peripheral blood by flow cytometry analysis. RO of responders (CR + PR + SD) and non-responders (PD) are shown. **b** The frequency of activated CD8+ T cells (CD3+ CD8+ CD45RA−) over time are shown in clinical responders and non-responders

In this study, PD-1 RO was determined as the percentage of receptor bound JS001 to total PD-1 on human whole blood lymphocytes. Receptor bound JS001 was detected by an isotype-specific antibody that recognizes the IgG_4_ Fc portion of JS001. Total PD-1 was assessed by first incubating the cells with a saturating concentration of JS001 and then staining the cells with a PE-labeled anti-human IgG_4_Fc. JS001^+^ target cells were predominantly present in the CD45RA^−^ T cell subset, which represents non-naïve or an activated T cell population. As shown in Additional file 1: Table S3, the majority of subjects in the 3 dose cohorts maintained full occupancy (> 80%) of the PD-1 receptor on the peripheral blood T lymphocytes over the course of treatment. As shown in Fig. 3a, the pharmacodynamic readout of the PD-1 RO had no correlation with clinical response, as responders (CR + PR + SD) and non-responders (PD) had similar RO during treatment.

The frequency and activation status (by CD45RA staining) of T lymphocytes in whole blood were also evaluated during the course of treatment. There was no major impact of JS001 on the frequency of activated CD8+ T lymphocytes in the peripheral blood in individual subjects (Additional file 1: Figure S3). Interestingly, when the percentage of activated CD8+ T cells of clinical responders (CR + PR + SD) and non-responders were compared, subjects with high percentage of activated CD8+ cells were enriched in the group with a PFS greater than 250 days (Fig. 3b). While the average percentage of activated CD8+ cells in all subjects was 65% before JS001 treatment, 4 out of 5 durable responders (PFS > 300 days) had activated a CD8+ percentage over 80% (of all CD8+ cells), indicating a potential correlation of favorable response to immunotherapy, with high percentage of activated CD8 cells in the peripheral blood prior to treatment. Similarly, subjects with a higher percentage of CD3− CD16+ CD54+ natural killer cells (≥ 26% of all lymphocytes) at baseline (*n* = 10) also responded better than subjects with less than 26% NK cells (*n* = 26) (40% ORR and 60% DCR versus 15.4% ORR and 46.2% DCR). The same 4 durable responders (PFS > 300 days) also had a starting NK cell percentage over 26% in the blood.

### Biomarker and subgroup analysis for correlation with clinical efficacy

Previous studies have shown the PD-1 pathway blockade may be particularly effective in melanoma, NSCLC, and other solid tumors with abundant TILs and high PD-L1 expression [22], regardless of its histology origin. PD-L1 CD8 double IHC staining was performed on available subject tumor biopsy samples (*n* = 28) in this study. Spring Bioscience (Roche) rabbit anti-human PD-L1 mAb clone SP142 [23] and anti-CD8 clone 4B11 were used. PD-L1-positive status was defined as the presence of membrane staining of any intensity in ≥ 1% of tumor cells in this study.

**Fig. 4:**
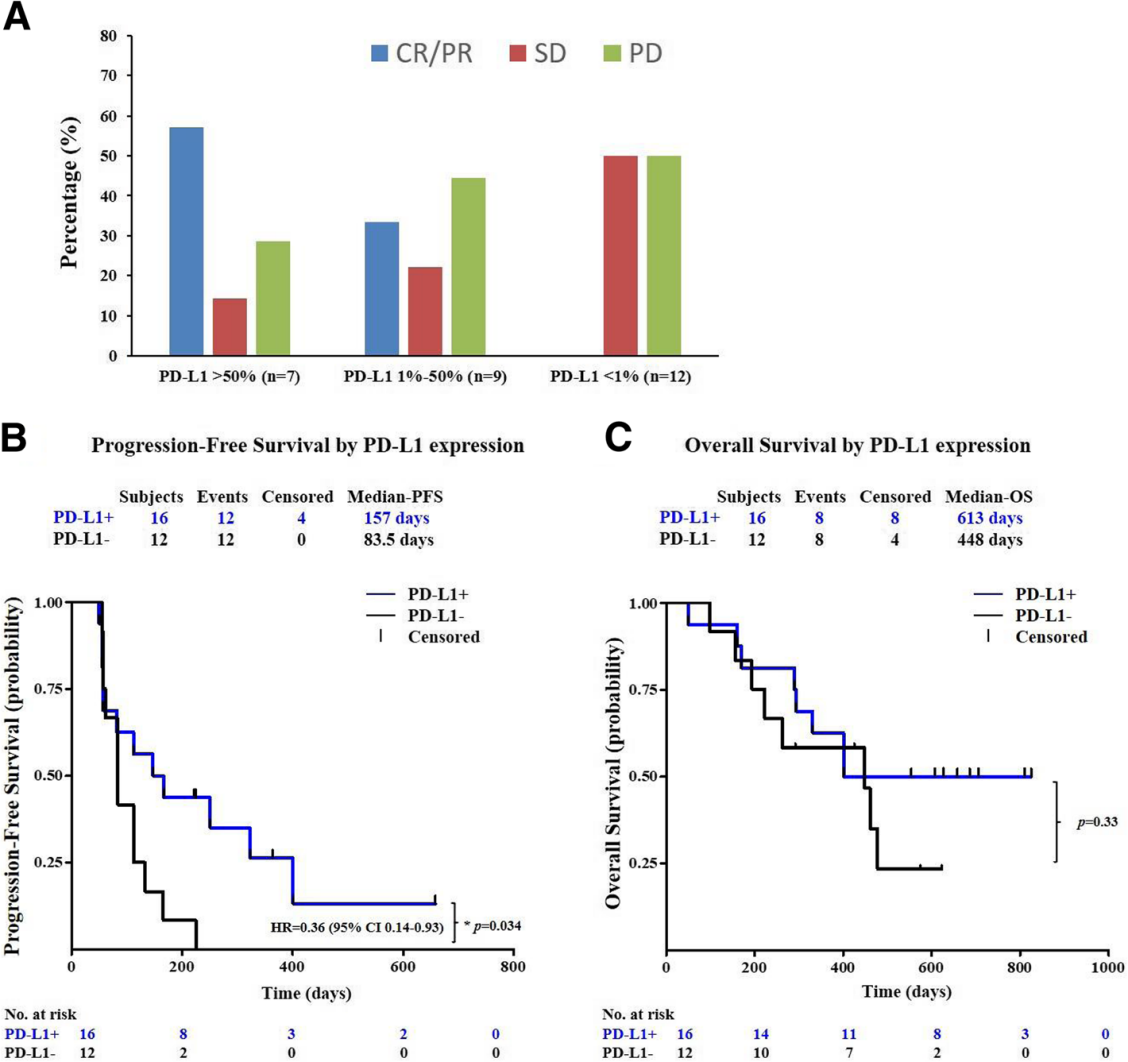
Correlation of PD-L1 expression by IHC staining in tumor biopsy with clinical efficacy. **a** High (> 50%) (*n* = 7), medium (1–50%) (*n* = 9), and low (< 1%) (*n* = 12) PD-L1 expression subgroups determined by anti-PD-L1 (SP142) IHC staining on tumor cells are compared for clinical response. **b** PFS of subjects by PD-L1 expression. PD-L1+ subjects showed significant PFS benefit than PD-L1− subjects, HR = 0.36 (95% CI 0.14–0.93), *p* = 0.034. **c** OS of subjects by PD-L1 expression. Percentages of survival patients are shown at indicated time points. Numbers of patients at risk at indicated time points are shown below the *x*-axis

**Fig. 5:**
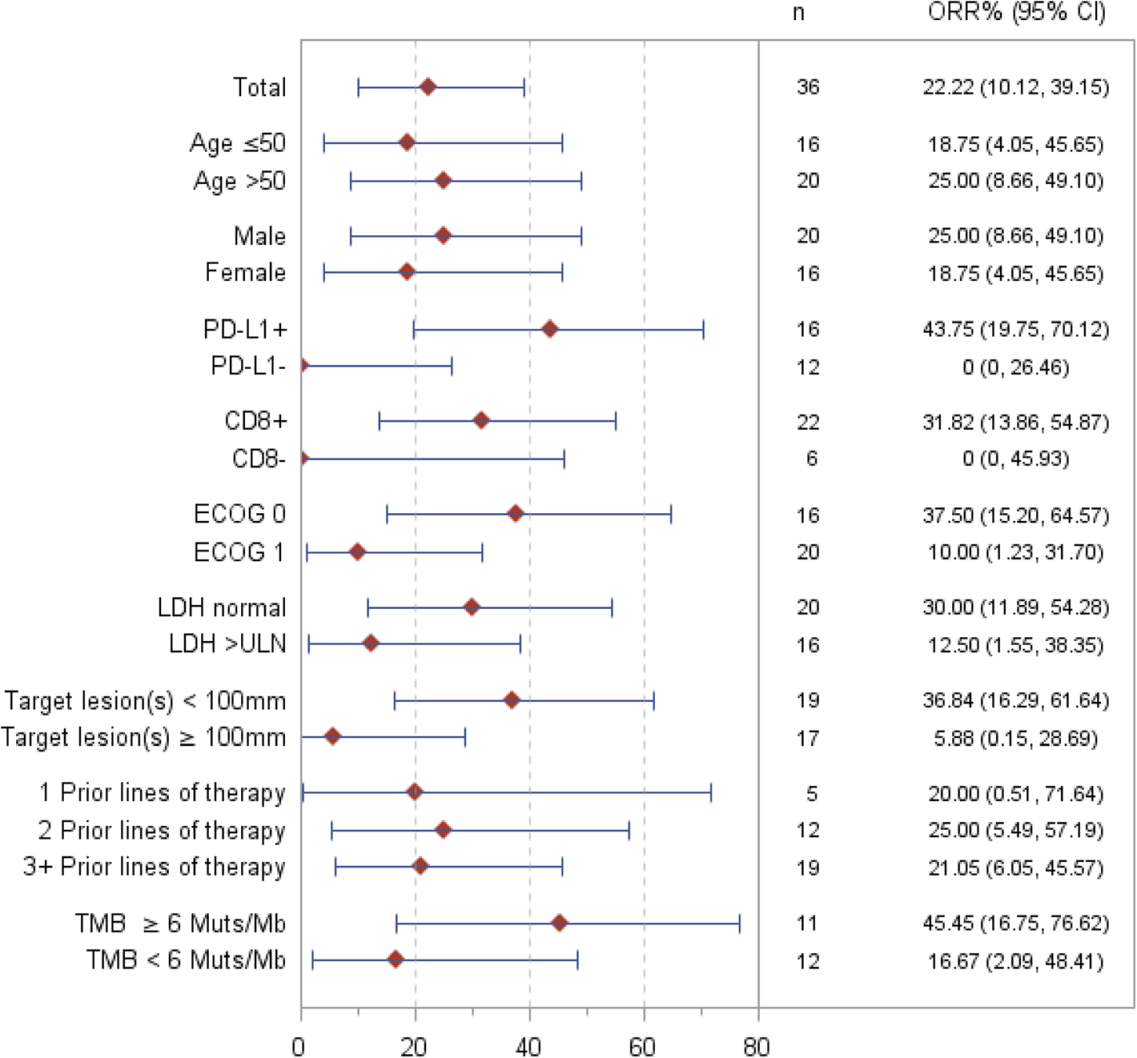
Correlation of biomarkers or subgroups with clinical efficacy. Subgroups by age, gender, PD-L1 expression on tumor cells, the presence of CD8+ TILs, ECOG performance score, LDH serum levels, baseline tumor burden, and prior lines of treatment are compared for clinical response to JS001 treatment. PD-L1-positive status was defined as the presence of membrane staining of any intensity in ≥ 1% of tumor cells by SP142 IHC staining. Upper limit of normal (ULN) for LDH serum level is 250 U/L. Baseline tumor burden was represented by sum of diameters of target lesion(s) per RECISTv1.1, and 100 mm was used as a cutoff

**Fig. 6:**
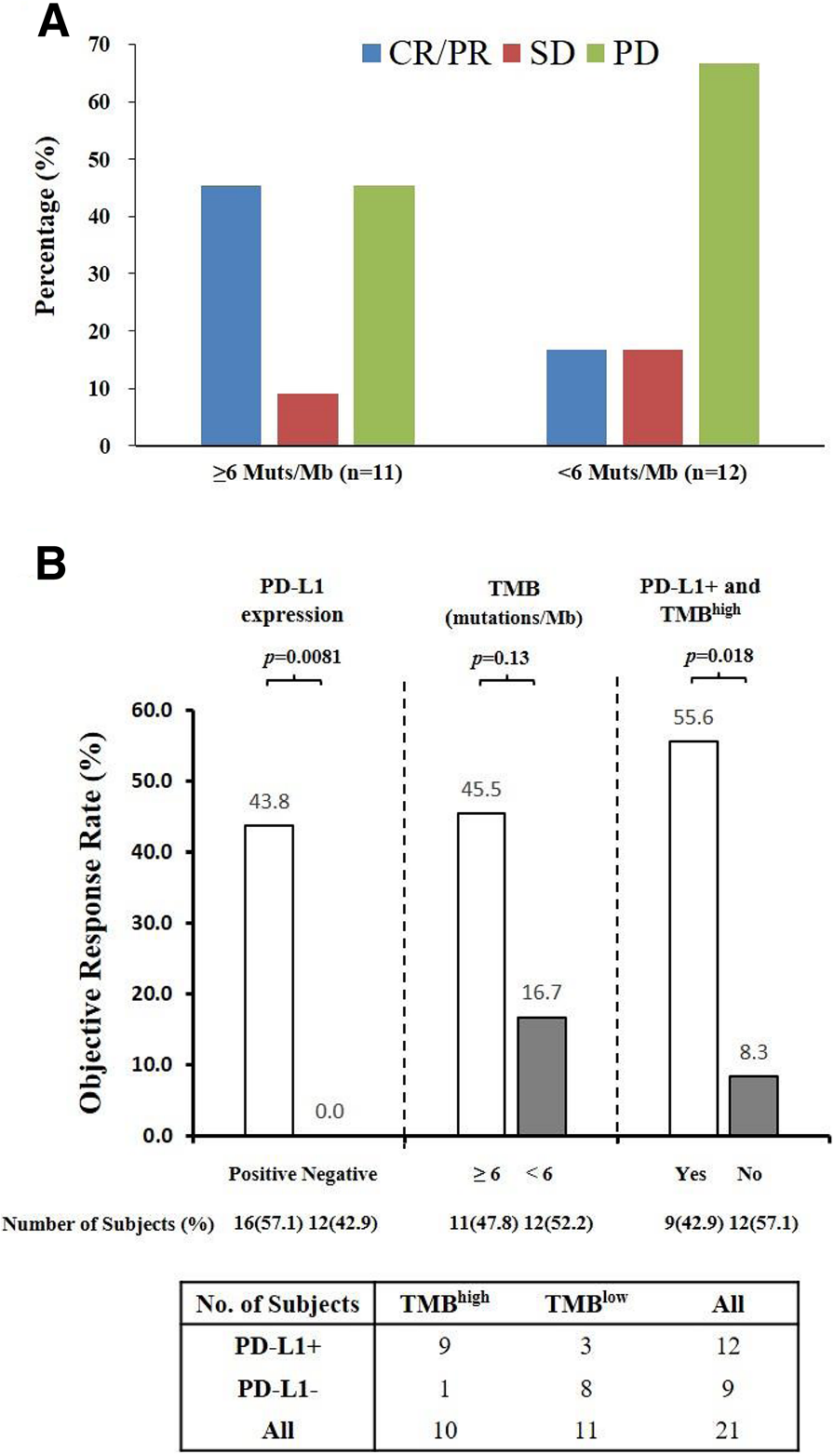
Correlation of TMB with objective response and overall survival upon JS001 treatment. Genomic profiling was performed by next-generation sequencing with a 450 cancer-related gene panel on both FFPE tumor and paired peripheral blood samples from 23 available subjects. The TMB was calculated by summing up somatic mutations within the coding regions examined the NGS panel. **a** Correlation of TMB with clinical response. Six mutations per Mb was used as a cutoff. **b** Correlation of TMB, PD-L1 status, and clinical response

As shown in Fig. 4a, subjects with PD-L1-positive expression in their tumor biopsy benefited more from JS001 treatment than PD-L1-negative subjects (43.8% ORR versus 0% ORR, *p* = 0.0081) (Fig. 6b). The PD-1 expression high subgroup (> 50%) responded most favorably to JS001 treatment, with a 57.1% ORR and a 71.4% DCR. Furthermore, PD-L1+ subjects showed significant PFS benefit than PD-L1− subjects from JS001 treatment, HR = 0.36 (95% CI 0.14–0.93), *p* = 0.034 (Fig. 4b), and numerically better median OS, 613 days versus 448 days (Fig. 4c). But the OS difference was not statistically significant, *p* = 0.33. Notably, all CR/PR subjects were PD-L1 and TIL double positive. Co-localization of TILs and PD-L1+ cells were observed in tumor biopsies as 93.8% (15/16) PD-L1-positive tumors also harbored TILs. Consistently, TIL-positive subjects responded numerically better to JS001 treatment than TIL-negative subjects (31.8% ORR versus 0% ORR), but the difference was not statically significant (*p* = 0.11) presumably due to small sample size (Fig. 5). Nevertheless, PD-L1-negative and TIL-negative subjects still benefited from PD-1 blockade therapy as 50% of either group achieved stable disease after JS001 treatment.

Additional biomarkers or parameters analyzed for correlation with clinical efficacy included age, gender, baseline tumor burden, prior lines of treatment, ECOG status, and serum LDH levels (Fig. 5). Among the subgroups, ECOG performance status of 0, serum LDH level within normal range and low tumor volume (the sum of target lesion(s) diameter < 100 mm) at baseline had numerically better clinical response to JS001 treatment. However, only subjects with baseline low tumor burden (< 100 mm) had statistically better response than subjects with high tumor burden (≥ 100 mm), 36.8% ORR versus 5.9% ORR, *p* = 0.026 (Fig. 6 and Additional file 1: Table S4).

In summary, favorable clinical responses were correlated with PD-L1 expression on tumor cells, the presence of tumor infiltrating lymphocytes, ECOG status at 0, serum LDH in normal range, and low tumor volume prior to treatment.

### Genomic profiling and tumor mutational burden measurement

Recent studies have found high TMB often correlated with a pre-existing adaptive immune response against the tumor and enhanced clinical efficacy in response to PD-1 blockade immunotherapy [24, 25]. Comprehensive genomic profiling was performed in this study by next-generation sequencing with a 450 cancer-related gene panel [21] (Yuansu Cancer Panel, OrigiMed) on both FFPE tumor and paired peripheral blood samples from 23 available subjects. The TMB was estimated by analyzing somatic mutations within the coding region of the cancer genome covering by the panel. As shown in Additional file 1: Table S5, the TMB was fewer than 10 mutations per Mb for all subjects expect one UC subject (152.8 Muts/Mb) and one acral melanoma subject (17.6 Muts/Mb), consistent with previous reports that acral and mucosal melanoma had limited DNA mutational burden [18] [26]. Higher TMB levels appear to be correlated with better clinical response (Fig. 6). Multiple potential cutoff points were evaluated, 6 mutations per MB provided the most numeric difference in response rate between CR/PR and PD groups. Subjects with 6+ Muts/Mb (*n* = 11) achieved numerically better clinical response than subjects with fewer than 6 Muts/Mb (*n* = 12) (45.5% ORR and 54.5% DCR versus 16.7% ORR and 33.3% DCR) (Fig. 6a); however, the difference was not statically significant, *p* = 0.13 (Fig. 6b). TMB^high^ and PD-L1+ populations were largely overlapping in this study. From 21 subjects with valid results in both TMB and PD-L1 expression, 9 out of 10 TMB^high^ subjects were also PD-L1+, while 9 out of 12 PD-L1+ subjects were also TMB^high^.

**Fig. 7:**
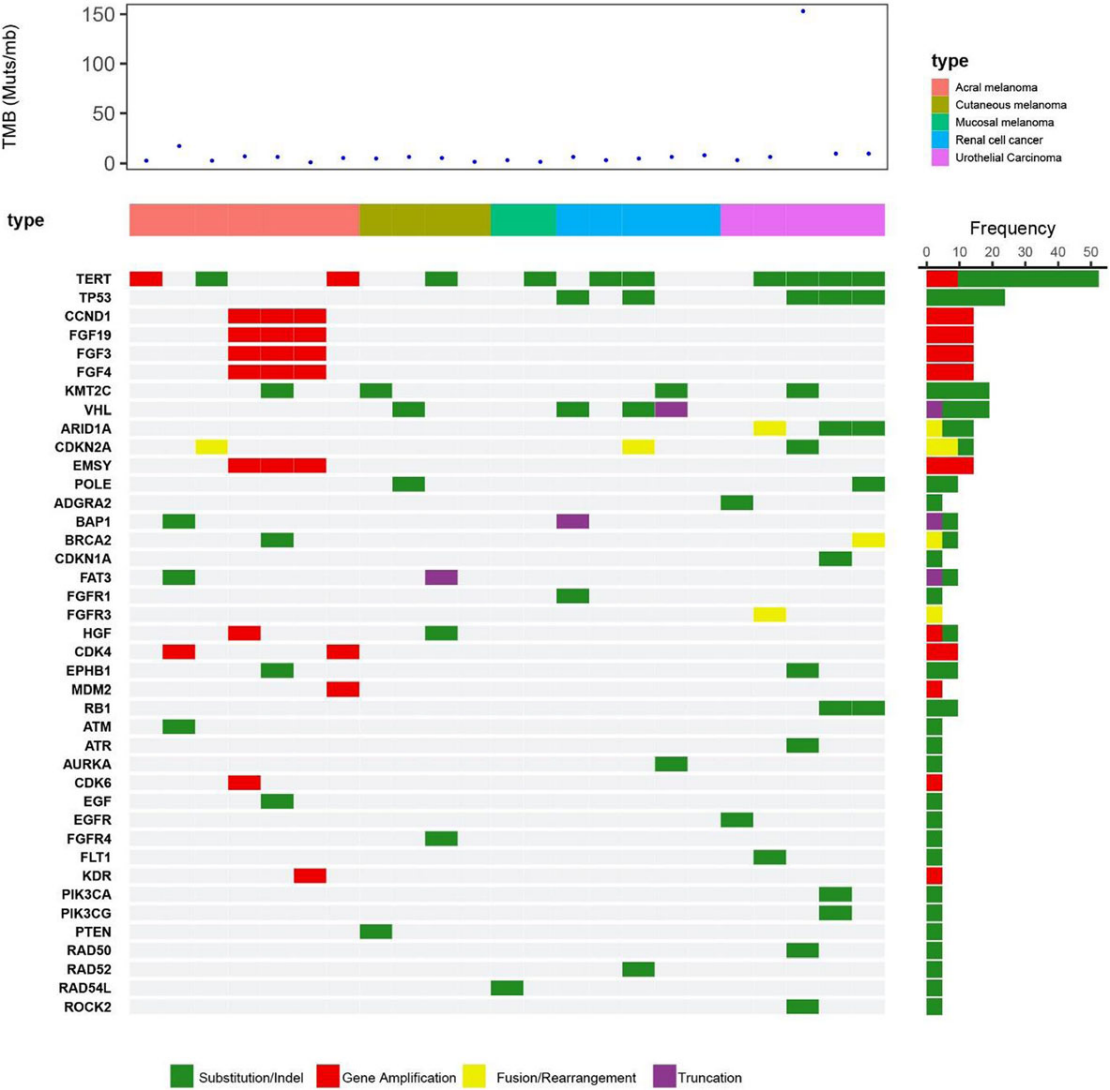
Genomic profiling of enrolled subjects. Genomic profiling was performed by next-generation sequencing with a 450 cancer-related gene panel on both FFPE tumor and paired peripheral blood samples from 23 available subjects. Genomic alterations including SNV, short and long INDELs, CNV, and gene rearrangement and fusions were assessed

Genomic profiling also identified frequent gene amplifications in available acral melanoma subjects (*n* = 7) in the study, including cell cycle regulators (Cyclin D1, CDK4, or CDK6) (5 out of 7), and genes locating in 11q13 such as fibroblast growth factors (FGF19, FGF3, and FGF4) and EMSY (a BRCA2 inactivating gene) (3 out of 7) (Fig. 7). Such clustered gene amplification was not observed in mucosal or UV-induced cutaneous melanoma, UC, or RCC subjects, suggesting a divergent tumorigenesis mechanism for the acral melanoma subtype. In comparison, RCC and UC tumors commonly harbor missense mutations in TP53 (5 out of 10) and SNVs at two hotspots in TERT promoter at nucleotide positions − 124 and − 146 upstream of ATG upregulating telomerase mRNA expression (6 out of 10) (Fig. 7), which occurred much less frequently in acral melanoma subjects in this study (0 out 7 for P53 mutation and 1 out of 7 for TERT promoter hotspots).

## Discussion

In recent years, the regulatory approvals and successful application of PD-1 pathway blockade treatment in a variety of solid tumors has highlighted the power of immunotherapy in controlling not only the classic immunogenic tumors, such as melanoma and RCC, but also malignancies historically considered resistant to immunotherapy such as NSCLC. The activity spectrum of PD-1 blockade was explored in this phase I dose escalation study with JS001, a new humanized IgG_4_ PD-1 mAb for treating acral (*n* = 14) and mucosal (*n* = 4) melanomas, together with RCC and UC subjects.

Unlike chronic UV exposure induced cutaneous melanoma, both acral and mucosal melanoma subtypes harbor much less DNA mutations [18] and were more refractory to standard chemotherapy as well as immunotherapy alone [27]. In sharp contrast to the dominance of chronic-sun-damage (CSD) type melanoma in the western world (95% of all melanomas), acral and mucosal melanoma are the two most common melanoma types in Asia, accounting for over 70% of melanomas in that region. Previous melanoma immunotherapy research has not focused on these two distinctive melanoma subtypes with drastically different underlying disease mechanisms. In the current study, we prospectively enrolled acral and mucosal melanoma subjects consistent with the regional epidemiology and evaluated their response to PD-1 blockade immunotherapy.

In summary, JS001 had an acceptable overall safety profile up to 10 mg/kg Q2W with no novel safety signal identified. Confirmed objective clinical responses were observed in every dose level of 1, 3, and 10 mg/kg Q2W, and in both acral (1 CR and 2 PR) and mucosal (1 PR) melanomas. The response was durable in subjects who were heavily pretreated (2 to 5 lines of prior systemic treatment). JS001 was particularly effective in subjects with high PD-L1 expression and abundant TILs, regardless of tumor histology subtypes, indicating the pre-existing T cell response against tumors in the microenvironment was the predominant determinant for response to PD-1 blockade therapy. Consistently, higher TMB was also correlated with better clinical response (using 6 mutations/Mb as a cutoff). TMB was generally low for acral and mucosal melanoma subjects in the study (all except one had less than 10 mutations per Mb), consistent with previous reports. TMB^high^ and PD-L1+ populations were largely overlapping in this study. From 21 subjects with valid results in both TMB and PD-L1 expression, 9 out of 10 TMB^high^ subjects were also PD-L1+, while 9 out of 12 PD-L1+ subjects were also TMB^high^. For the double positive group, 1 CR, 4 PR, 1 SD (unconfirmed PR), and 3 PD were observed. The PD-L1+ and TMB^high^ double positive group (*n* = 9) responded statistically better than the PD-L1− or TMB^low^ group (*n* = 12) to JS001 treatment, 55.6% ORR versus 8.3% ORR, *p* = 0.018 (Fig. 6b). However, PD-L1 expression was better than gene panel-derived TMB in predicting clinical response in this study, as only PD-L1-positive subjects had statically better survival benefits in PFS, HR = 0.36 (95% CI 0.14–0.93), *p* = 0.034 (Fig. 4 and Additional file 1: Figure S4).

Additional subgroup analysis found the general immune-competent status of a subject, including ECOG status, serum LDH levels, and baseline tumor volume which also had numerically better clinical response, whereas age, gender, and prior lines of treatment did not appear to play significant roles. Interestingly, 4 out of 5 durable responders (PFS > 300 days) had over 80% activated CD8+ T cells (of total CD8 T cells) and over 26% CD3− CD16+ CD54+ NK cells (of total lymphocytes) in the peripheral blood, whereas the average percentage of activated CD8+ T cells and NK cells in all subjects were 65% and 20% respectively, suggesting a potential favorable response to immunotherapy for subjects with a high percentage of activated CD8+ T cells and NK cells in circulation prior to treatment.

Genomic profiling of 23 available subjects also identified gene amplifications of cell cycle regulators (Cyclin D1, CDK4, or CDK6), fibroblast growth factors (FGF19, FGF3, and FGF4) and EMSY (a DNA repair inactivating gene) uniquely clustered in acral melanoma subjects in the study. Interestingly, 2 out of 7 acral melanoma subjects responded to JS001 treatment (1CR and 1PR), and both harbored EMSY gene amplification, which was suggested to be functionally equivalent to somatic BRCA2 loss-of-function mutations [28]. These two subjects were also classified into the TMB high group in this study (higher than 6 mutations/Mb). Further correlation of EMSY gene amplification with response to PD-1 blockade needs to be investigated in a larger cohort of acral melanoma subjects. Genomic profiling in this study has thus confirmed the previous finding of frequent aberration in the CDK4 pathway of acral melanoma subjects [29] and suggested a combination potential of CDK4/6 inhibitor, FGF/FGFR inhibitor, or DNA repair targeting agent such as PARP inhibitor with PD-1 blockade in treating acral melanomas. In contrast, mucosal melanoma subjects had limited DNA mutations or gene amplification in this study. VEGF overexpression and the density of micro-vessel formation were associated with poor prognosis in mucosal melanoma [30]. The combination of VEGF inhibition with PD-1 blockade might hold the key to enhance the response of mucosal melanoma to immunotherapy.

PD-1 blockade-based immunotherapy has drastically improved the survival of late stage metastatic cancer patients who had limited therapeutic options in recent years. It remains a challenge to further improve the response rate beyond the current ceiling of an average 20–30 percentile. Using melanoma as an example, biomarker-guided divergent combination strategies to treat different subtypes could be envisioned. We look forward to validating our findings in the ongoing registration trials of JS001 for 2nd line (NCT03013101) and 1st line (NCT03430297) treatment of metastatic melanoma subjects with emphasis on acral and mucosal subtypes.

## Conclusions

JS001 was well tolerated in humans, with mainly grade 1 or grade 2 TEAEs. In this study, JS001 has demonstrated promising durable anti-tumor activity in UC, RCC as well as in previously underexplored acral (*n* = 13, 21.4% ORR, 42.9% DCR) and mucosal (*n* = 4, 25% ORR, 50% DCR) melanoma subtypes, which account for over 70% of melanoma patients in Asia and are not caused by chronic ultraviolet (UV) exposure. Subjects with an immune-active profile in the tumor microenvironment (PD-L1+, TIL+, and TMB high) or in peripheral blood (high percentage of activated CD8+ T cells and NK cells) responded favorably to JS001 treatment. The completion of the current phase I study has led to the initiation of the first prospective anti-PD-1 registration trial in Asia focusing on acral and mucosal melanoma subtypes, representative of the regional disease epidemiology.

## Additional file

Additional file 1: **Figure S1.** Phase I study schema of JS001 in advanced melanoma, renal cell carcinoma, and urothelial carcinoma. **Figure S2.** The PK profiles of JS001 in humans. **Figure S3.** The percentage of activated CD8+ T cell population during JS001 treatment. **Figure S4.** Correlation of tumor mutational burden (TMB) with clinical efficacy. **Table S1.** Treatment-related serious adverse events (SAE). **Table S2.** Grade 3 and above treatment-related adverse events (TRAE) in each cohort. **Table S3.** PD-1 receptor occupancy (RO) by JS001 in three dose cohorts. **Table S4.** Subgroup analysis of correlation with clinical efficacy. **Table S5.** Tumor mutational burden measurement and correlation with clinical response. (DOCX 575 kb)

## Abbreviations

ADA: Anti-drug antibodies
AE: Adverse events
ALM: Lentiginous melanoma
CCP: Confirmation cut point
CDR: Complementary-determining regions
CHO: Chinese Hamster Ovary
*C*_max_: Maximum observed concentration
CNV: Copy number variants
CR: Complete response
CSD: Chronic sun damage
DBIL: Direct bilirubin
DCR: Disease control rate
DLT: Dose-limiting toxicity
ECL: Electrochemiluminescence
ECOG: Eastern Cooperative Oncology Group
FFPE: Formalin-fixed paraffin-embedded
FGF: Fibroblast growth factors
IHC: Immunohistochemistry
INDELs: Insertions/deletions
IP: Intraperitoneally
irAEs: Immune-related AEs
ITT: Intention-to-treat
IV: Intravenous
mAb: Monoclonal antibody
MM: Mucosal melanoma
MTD: Maximum-tolerated dose
ORR: Objective response rate
OS: Overall survival
PD-1: Programmed death-1
PD-L: PD-1 ligand
PFS: Progression-free survival
PK: Pharmacokinetics
PR: Partial response
RCC: Renal cell cancer
RECIST: Response Evaluation Criteria in Solid Tumors
RO: Receptor occupancy
RT: Room temperature
SAE: Treatment-related serious adverse events
SCP: Screening cut point
SD: Stable disease
SNV: Single-base substitution
TIL: Tumor infiltrating lymphocyte
TMB: Tumor mutational burden
TRAE: Treatment-related adverse events
UC: Urothelial cancer
UV: Chronic ultraviolet
