# Supplementary material for: Safety and clinical activity with an anti-PD-1 antibody JS001 in advanced melanoma or urologic cancer patients
Source: J Hematol Oncol. 2019 Jan 14;12:7. doi: 10.1186/s13045-018-0693-2 (PMC6332582; doi:10.1186/s13045-018-0693-2)
Supplement: Supplementary file 1 — Figure S1. Phase I study schema of JS001 in advanced melanoma, renal cell carcinoma, and urothelial carcinoma. Figure S2. The PK profiles of JS001 in humans. Figure S3. The percentage of activated CD8+ T cell population during JS001 treatment. Figure S4. Correlation of tumor mutational burden (TMB) with clinical efficacy. Table S1. Treatment-related serious adverse events (SAE). Table S2. Grade 3 and above treatment-related adverse events (TRAE) in each cohort. Table S3. PD-1 receptor occupancy (RO) by JS001 in three dose cohorts. Table S4. Subgroup analysis of correlation with clinical efficacy. Table S5. Tumor mutational burden measurement and correlation with clinical response. (DOCX 575 kb) [file 13045_2018_693_MOESM1_ESM.docx]

**Appendix Figures AND TABLES**

**Figure S1: Phase I study schema of JS001 in advanced melanoma, renal cell carcinoma and urothelial carcinoma.** MEL, melanoma. RCC, renal cell carcinoma. UC, urothelial carcinoma.CR, complete response. PR, partial response. SD, stable disease. PD, progressive disease.

**
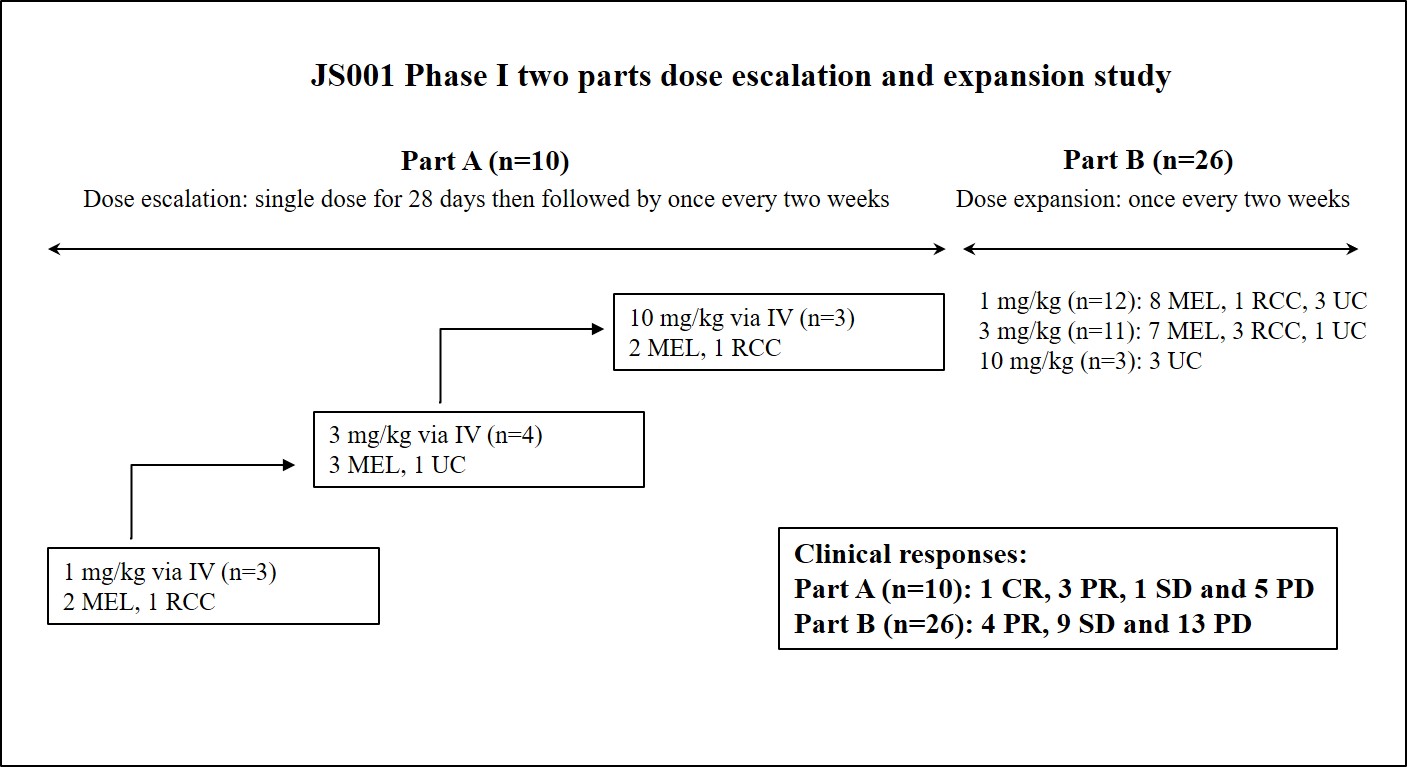
**

**Figure S2: The PK profiles of JS001 in humans.** **(A)** PK parameters of JS001 after a single dose for 28 days and multi-dose infusions every two weeks in three dose cohorts of 1 mg/kg, 3 mg/kg and 10 mg/kg. **(B)** Serum concentration of JS001 in three cohorts over 98 days.

| PK Parameter | Unit | 1 mg/kg |  | 3 mg/kg |  | 10 mg/kg |  |  |
| --- | --- | --- | --- | --- | --- | --- | --- | --- |
|  |  | JS001 |  | JS001 |  | JS001 |  |  |
|  |  | Mean ± SD | n | Mean ± SD | n | Mean ± SD | n |  |
| Day29 | | | | | | | | |
| Kel | 1/hr | 0.005±0.001 | 3 | 0.004±0.002 | 3 | 0.003±0.002 | 3 |  |
| t1/2 | hr | 149.56±30.93 | 3 | 185.05±84.91 | 3 | 235.68±98.52 | 3 |  |
| Tmax | hr | 0.5-6.0 | 3 | 0.5-2.0 | 3 | 0.5-2.0 | 3 |  |
| Cmax | μg/mL | 21.85±5.12 | 3 | 85.36±36.09 | 3 | 232.00±45.22 | 3 |  |
| AUC(0-t) | hr*μg/mL | 3263.11±320.28 | 3 | 8841.86±3671.44 | 3 | 36185.78±17056.22 | 3 |  |
| AUC(0-inf) | hr*μg/mL | 4143.19±154.40 | 3 | 13307.31±7564.85 | 3 | 65863.66±41568.62 | 3 |  |
| AUC(t-inf)% | % | 21.16±8.59 | 3 | 27.92±14.91 | 3 | 37.09±20.00 | 3 |  |
| Vd | mL/kg | 52.08±10.84 | 3 | 68.35±27.93 | 3 | 60.70±21.63 | 3 |  |
| Cl | mL/hr/kg | 0.24±0.01 | 3 | 0.30±0.22 | 3 | 0.24±0.21 | 3 |  |
| MRTinf | hr | 215.59±47.06 | 3 | 264.74±124.93 | 3 | 337.61±151.10 | 3 |  |
| Day113 | | | | | | | | |
| Kel | 1/hr | 0.003±0.001 | 3 | 0.002±0.001 | 2 | 0.002±0.001 | 2 |  |
| t1/2 | hr | 228.83±47.43 | 3 | 395.31±186.20 | 2 | 334.11±131.75 | 2 |  |
| Tmax | hr | 0.5-6.0 | 3 | 0.5 | 2 | 0.0 | 2 |  |
| Cmax | μg/mL | 47.05±16.97 | 3 | 105.19±32.67 | 2 | 323.77±41.96 | 2 |  |
| AUC(0-t) | hr*μg/mL | 5683.85±1285.99 | 3 | 19789.32±3974.65 | 2 | 56328.37±16800.53 | 2 |  |
| AUC(0-inf) | hr*μg/mL | 8815.79±2382.45 | 3 | 44022.34±6105.77 | 2 | 126194.03±72330.76 | 2 |  |
| AUC(t-inf)% | % | 34.44±9.16 | 3 | 53.98±15.41 | 2 | 51.16±14.68 | 2 |  |
| Cmin | μg/ml | 11.02±1.98 | 3 | 39.64±7.54 | 2 | 127.41±55.69 | 2 |  |
| Cavg | μg/ml | 16.89±3.82 | 3 | 61.81±11.98 | 2 | 176.36±54.16 | 2 |  |
| Fluctuation% | % | 206.20±57.58 | 3 | 104.08±20.47 | 2 | 118.10±44.06 | 2 |  |
| CLss | ml/hr/kg | 0.18±0.04 | 3 | 0.15±0.03 | 2 | 0.18±0.05 | 2 |  |
| MRTinf | hr | 314.81±80.95 | 3 | 541.63±247.74 | 2 | 491.48±210.36 | 2 |  |
| Vz | ml/kg | 59.36±14.51 | 3 | 87.80±55.83 | 2 | 80.20±7.45 | 2 |  |
| Vss | ml/kg | 56.74±17.25 | 3 | 83.28±51.94 | 2 | 81.32±10.52 | 2 |  |
| Accumulation_Index |  | 1.57±0.19 | 3 | 2.25±0.77 | 2 | 2.00±0.54 | 2 |  |
| AUC_TAU | hr*μg/ml | 5675.70±1284.05 | 3 | 20766.78±4026.93 | 2 | 59258.36±18198.68 | 2 |  |

(A)


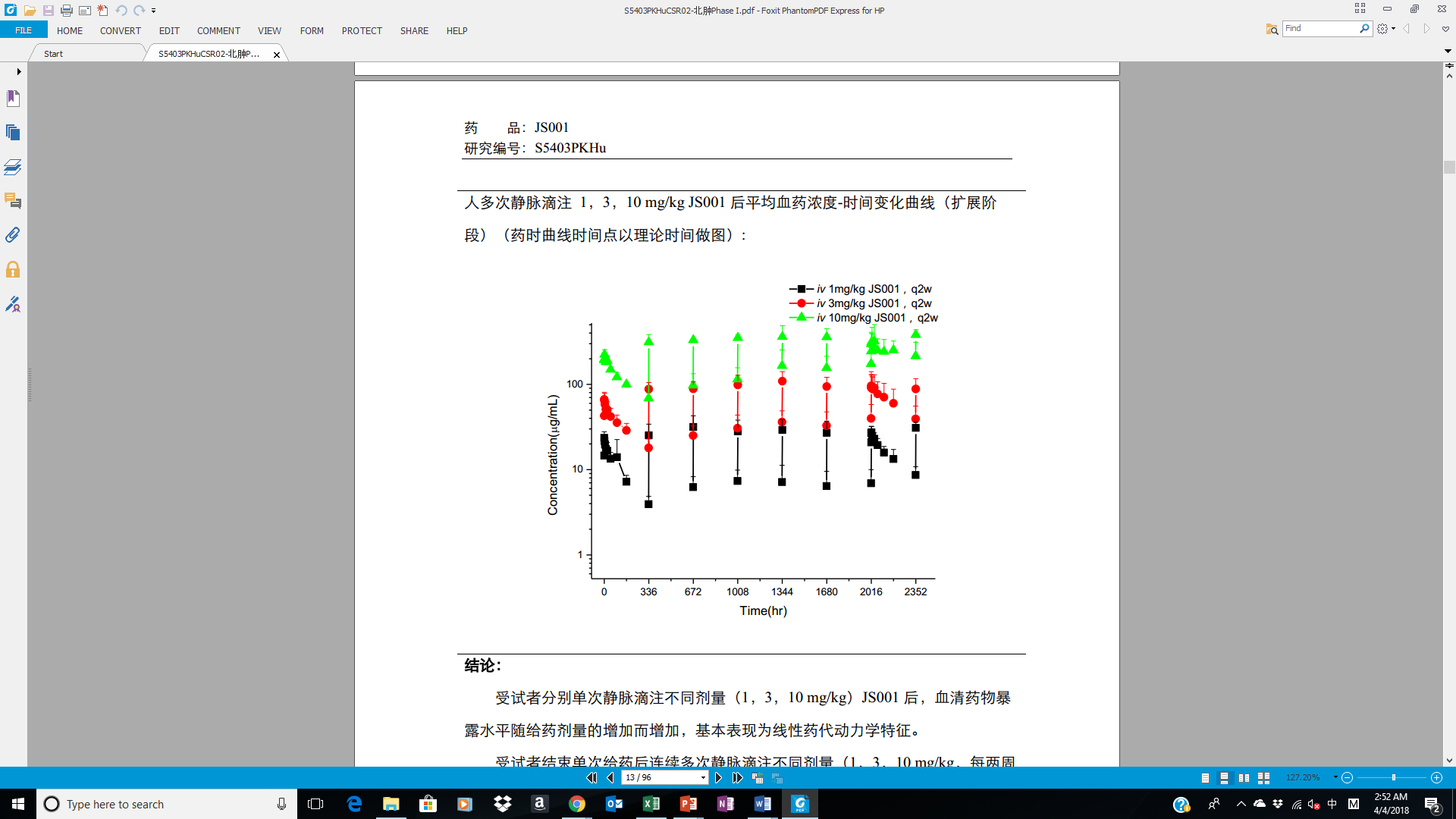
(B)

**Figure S3: The percentage of activated CD8+ T cell population during JS001 treatment.** No significant change was observed during the course of the study.


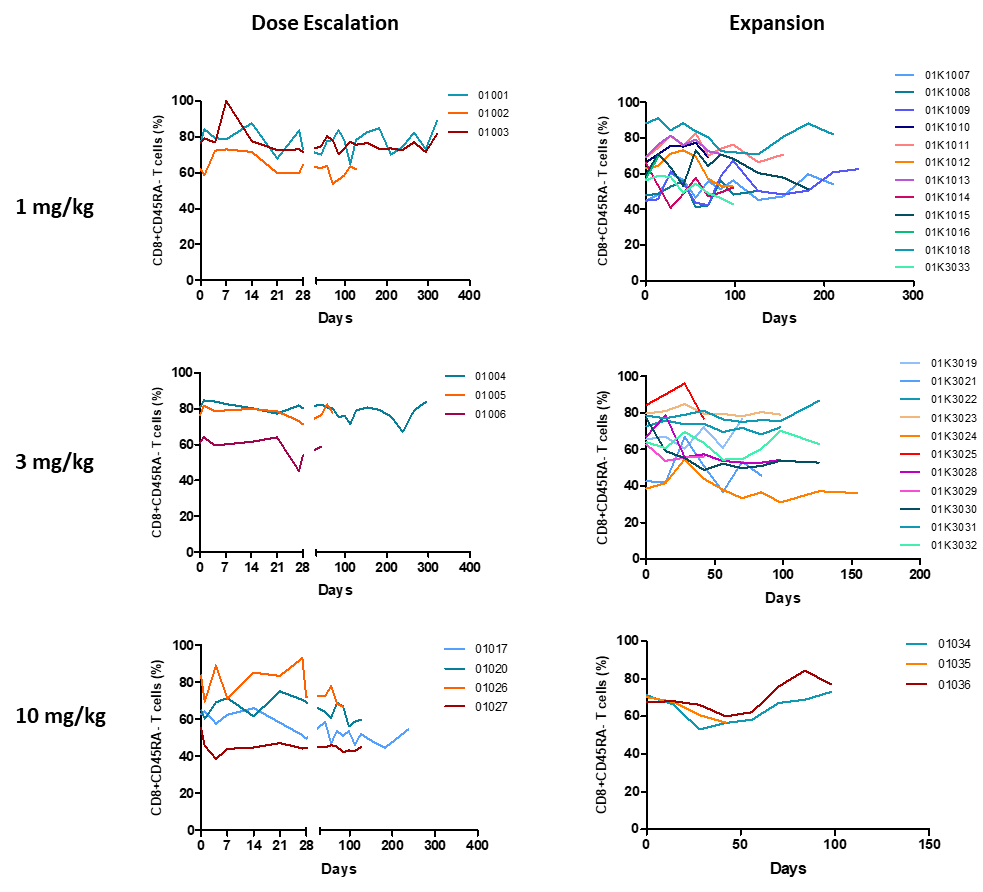


**Figure S4. Correlation of tumor mutational burden (TMB) with clinical efficacy. (A)** PFS of subjects by TMB≥6 Muts/Mb versus TMB <6 Muts/Mb. **(C)** OS of subjects by TMB≥6 Muts/Mb versus TMB <6 Muts/Mb. No difference was found between two groups. Percentages of survival patients are shown at indicated time points. Numbers of patients at risk at indicated time points are shown below the x-axis.


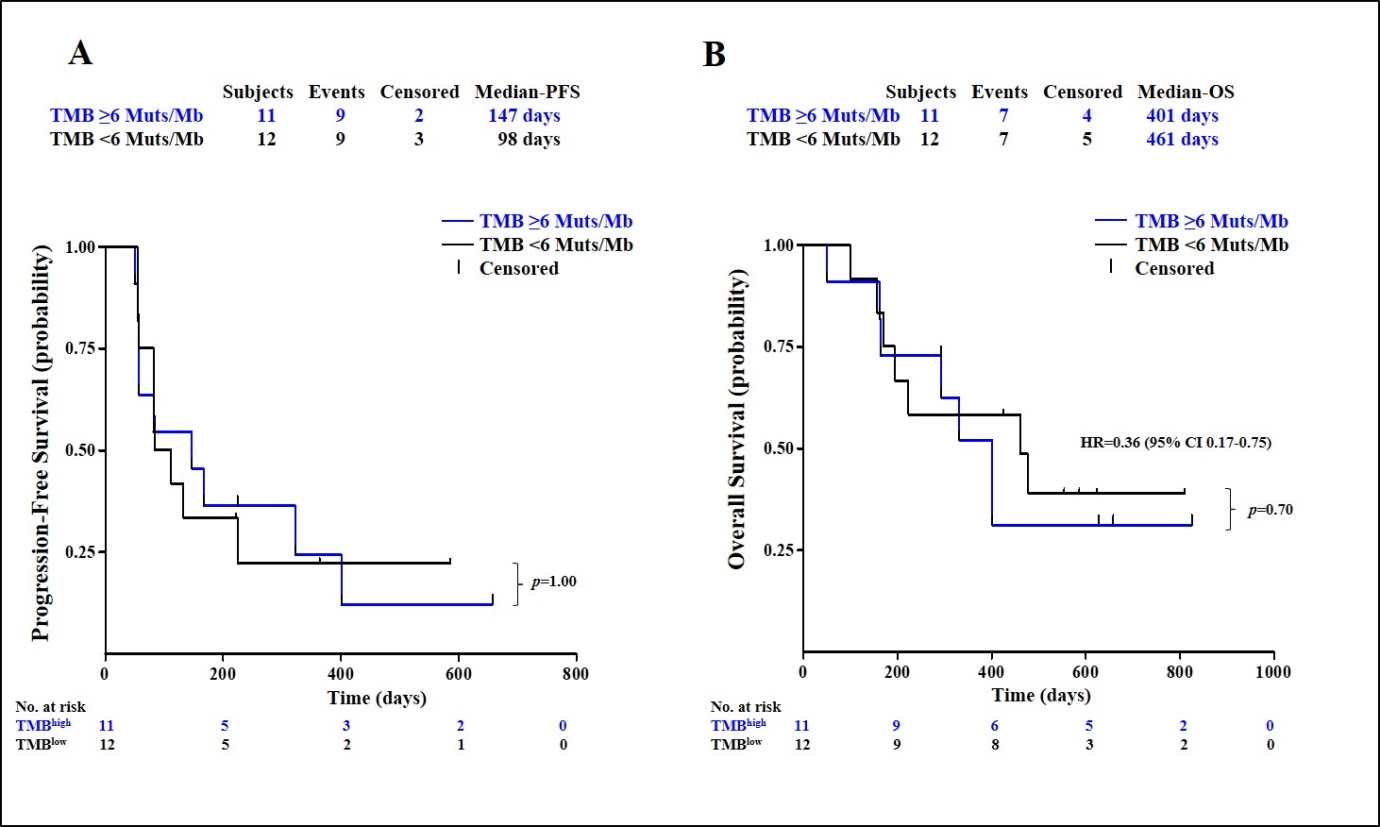


**Table S1. Treatment related serious adverse events (SAE).** By the safety data cut-off date of July 31 2018, treatment related SAEs and incidence rate in each cohort are listed.

|  | 1 mg/kg (n=15) | | 3 mg/kg (n=15) | | 10 mg/kg (n=6) | | Total (n=36) | |
| --- | --- | --- | --- | --- | --- | --- | --- | --- |
|  | Case | N (%) | Case | N (%) | Case | N (%) | Case | N (%) |
| Total | 3 | 2(13.33) | 1 | 1(6.67) | 3 | 2(33.33) | 7 | 5(13.89) |
| Appetite decreased | 0 | 0(0.00) | 0 | 0(0.00) | 2 | 1(16.67) | 2 | 1(2.78) |
| Lung infection | 0 | 0(0.00) | 1 | 1(6.67) | 0 | 0(0.00) | 1 | 1(2.78) |
| Disease progression | 0 | 0(0.00) | 0 | 0(0.00) | 1 | 1(16.67) | 1 | 1(2.78) |
| Fever | 1 | 1(6.67) | 0 | 0(0.00) | 0 | 0(0.00) | 1 | 1(2.78) |
| Irregular heart beat | 2 | 1(6.67) | 0 | 0(0.00) | 0 | 0(0.00) | 2 | 1(2.78) |

**Table S2. Grade 3 and above treatment related adverse events (TRAE) in each cohort.** By the safety data cut-off date of July 31 2018, treatment related grade 3 and above AE and incidence rate in each cohort are listed. G3, grade 3; G4, grade 4.

| Grade ≥3 TRAE | 1 mg/kg | G3 | G4 | 3 mg/kg | G3 | G4 | 10 mg/kg | G3 | G4 | Total | G3 | G4 |
| --- | --- | --- | --- | --- | --- | --- | --- | --- | --- | --- | --- | --- |
|  | N (%) |  |  | N (%) |  |  | N (%) |  |  | N (%) |  |  |
|  | 5(100.00) | 5 | 0 | 5(100.00) | 5 | 0 | 3(100.00) | 3 | 0 | 13(100.00) | 13 | 0 |
| Hypokalemia | 1(6.67) | 1 | 0 | 1(6.67) | 1 | 0 | 0(0.00) | 0 | 0 | 2(5.56) | 2 | 0 |
| ALT increased | 0(0.00) | 0 | 0 | 1(6.67) | 1 | 0 | 0(0.00) | 0 | 0 | 1(2.78) | 1 | 0 |
| Amylase increased | 0(0.00) | 0 | 0 | 0(0.00) | 0 | 0 | 1(16.67) | 1 | 0 | 1(2.78) | 1 | 0 |
| DBIL increased | 0(0.00) | 0 | 0 | 2(13.33) | 2 | 0 | 0(0.00) | 0 | 0 | 2(5.56) | 2 | 0 |
| Proteinuria | 1(6.67) | 1 | 0 | 0(0.00) | 0 | 0 | 0(0.00) | 0 | 0 | 1(2.78) | 1 | 0 |
| Creatine kinase increased | 1(6.67) | 1 | 0 | 0(0.00) | 0 | 0 | 0(0.00) | 0 | 0 | 1(2.78) | 1 | 0 |
| Serum Creatinine increased | 0(0.00) | 0 | 0 | 1(6.67) | 1 | 0 | 0(0.00) | 0 | 0 | 1(2.78) | 1 | 0 |
| Hyperglycemia | 0(0.00) | 0 | 0 | 0(0.00) | 0 | 0 | 1(16.67) | 1 | 0 | 1(2.78) | 1 | 0 |
| Low blood pressure | 0(0.00) | 0 | 0 | 1(6.67) | 1 | 0 | 0(0.00) | 0 | 0 | 1(2.78) | 1 | 0 |
| Lipase increased | 1(6.67) | 1 | 0 | 1(6.67) | 1 | 0 | 2(33.33) | 2 | 0 | 4(11.12) | 4 | 0 |
| Kidney disease | 1(6.67) | 0 | 0 | 1(6.67) | 1 | 0 | 0(0.00) | 0 | 0 | 1(2.78) | 1 | 0 |
| Anemia | 2(13.33) | 2 | 0 | 0(0.00) | 0 | 0 | 1(16.67) | 1 | 0 | 3(8.34) | 3 | 0 |

**Table S3. PD-1 receptor occupancy (RO) by JS001 in three dose cohorts.** The mean, median, SD and range of RO of activated T cells (CD3+ CD45RA-), activated CD8 T cells (CD3+ CD8+ CD45RA-) and activated CD4 T cells (CD3+ CD8- CD45RA-) are shown.

| **RO (%)** | **Activated T cell** | | | | **Activated CD8 T cell** | | | | **Activated CD4 T cell** | | | |
| --- | --- | --- | --- | --- | --- | --- | --- | --- | --- | --- | --- | --- |
| **Cohort** | **Mean** | **SD** | **Median** | **Range** | **Mean** | **SD** | **Median** | **Range** | **Mean** | **SD** | **Median** | **Range** |
| 1 mg/kg (n=13) | 91.66 | 8.59 | 91.91 | 65-100 | 89.68 | 7.35 | 90.16 | 70-100 | 93.04 | 9.65 | 95.44 | 62-100 |
| 3 mg/kg (n=14) | 95.21 | 6.29 | 97.08 | 79-100 | 92.97 | 6.87 | 93.80 | 75-100 | 96.38 | 5.26 | 98.66 | 82-100 |
| 10 mg/kg (n=7) | 97.52 | 1.78 | 96.65 | 95-100 | 95.12 | 4.02 | 95.32 | 88-99 | 98.24 | 1.23 | 97.46 | 97-100 |

**Table S4.** **Subgroup analysis of correlation with clinical efficacy.** Parameters analyzed for correlation with clinical efficacy included PD-L1 expression and TIL in tumor biopsy, ECOG performance score, LDH level, tumor burden at baseline, age, gender, prior lines of treatment. * PD-L1 positive status was defined as the presence of membrane staining of any intensity in ≥ 1% of tumor cells. ** ULN, upper limit of normal for LDH serum level, 250 U/L. *** Tumor volume was represented by sum of diameters of target lesions.100 mm was used as a cut-off.

| **Characteristic** | **Value** | **n** | **ORR** | **DCR** |
| --- | --- | --- | --- | --- |
| **Age** | **≤50** | 16 | 18.8% | 50.0% |
|  | **>50** | 20 | 25.0% | 50.0% |
| **Gender** | **Male** | 20 | 25.0% | 50.0% |
|  | **Female** | 16 | 18.8% | 50.0% |
| **PD-L1 expression** | **+** | 16 | 43.8% | 62.5% |
|  | **-** | 12 | 0.0% | 50.0% |
| **TIL** | **+** | 22 | 31.8% | 59.1% |
|  | **-** | 6 | 0.0% | 50.0% |
| **ECOG** | **0** | 16 | 37.5% | 56.3% |
|  | **1** | 20 | 10.0% | 45.0% |
| **LDH** | **normal** | 20 | 30.0% | 50.0% |
|  | **>ULN** | 16 | 12.5% | 50.0% |
| **Tumor volume** | **<100 mm** | 19 | 36.8% | 68.4% |
|  | **≥100 mm** | 17 | 5.9% | 29.4% |
| **Prior lines of therapy** | **1** | 5 | 20.0% | 60.0% |
|  | **2** | 12 | 25.0% | 58.3% |
|  | **3+** | 19 | 21.1% | 36.8% |
| **Total** |  | 36 | 22.2% | 50.0% |

**Table S5. Tumor mutational burden measurement and correlation with clinical response.** * Subjects were still alive on July 3, 2018 and OS was not reached for these subjects.

| **Subject** | **Type** | **TMB value (Muts/Mb)** | **PFS (days)** | **OS (days)** | **Response** |
| --- | --- | --- | --- | --- | --- |
| 01001* | Acral Melanoma | 6.40 | 323 | 825 | PR |
| 01002 | Acral Melanoma | 5.40 | 84 | 194 | PD |
| 01003* | RCC | 4.80 | 364 | 810 | SD |
| 01004 | Acral Melanoma | 7.20 | 401 | 401 | CR |
| 01005 | Melanoma | 6.40 | 82 | 161 | PD |
| 01006 | UC | 9.60 | 50 | 50 | PD |
| 01K1007 | UC | 9.60 | 167 | 401 | SD/uPR |
| 01K1008 | Acral Melanoma | 0.80 | 57 | 425 | PD |
| 01K1011 | Acral Melanoma | 2.40 | 112 | 478 | PD |
| 01K1012 | RCC | 6.20 | 57 | 293 | PD |
| 01K1013 | Melanoma | 4.80 | 133 | 156 | PD |
| 01K1014 | Acral Melanoma | 17.60 | 57 | 292 | PD |
| 1018* | UC | 152.80 | 658 | 658 | PR |
| 01K3019 | Mucosal Melanoma | 5.40 | 55 | 170 | PD |
| 01020* | RCC | 6.20 | 147 | 627 | PR |
| 01K3023* | RCC | 3.20 | 55 | 623 | PD |
| 01K3024 | Melanoma | 1.60 | 225 | 461 | SD |
| 01026 | Acral Melanoma | 2.40 | 83 | 99 | PD |
| 01027 | Melanoma | 1.60 | 83 | 223 | PD |
| 01K3030 | RCC | 7.80 | 225 | 331 | PR |
| 01K3032* | Mucosal Melanoma | 3.10 | 586 | 586 | PR |
| 01035 | UC | 6.40 | 55 | 163 | PD |
| 01036* | UC | 3.20 | 222 | 554 | PR |
